# Exosomal long noncoding RNA MLETA1 promotes tumor progression and metastasis by regulating the miR-186-5p/EGFR and miR-497-5p/IGF1R axes in non-small cell lung cancer

**DOI:** 10.1186/s13046-023-02859-y

**Published:** 2023-10-26

**Authors:** Xiu-Rui Hsu, Jia-En Wu, Yi-Ying Wu, Sheng-Yen Hsiao, Jui-Lin Liang, Ya-Ju Wu, Chia-Hao Tung, Meng-Fan Huang, Ming-Shiu Lin, Pan-Chyr Yang, Yuh-Ling Chen, Tse-Ming Hong

**Affiliations:** 1 Institute of Clinical Medicine, College of Medicine, National Cheng Kung University, Tainan, Taiwan; 2 Clinical Medicine Research Center, National Cheng Kung University Hospital, College of Medicine, National Cheng Kung University, Tainan, Taiwan; 3 Department of Internal Medicine, Division of Hematology-Oncology, Chi Mei Medical Center, Liouying, Tainan, Taiwan; 4 Department of Surgery, Chi-Mei Medical Center, Liouying, Tainan, Taiwan; 5 Department of Pathology, Chi Mei Medical Center, Liouying, Tainan, Taiwan; 6 Institute of Biomedical Sciences, Academia Sinica, Taipei, 115 Taiwan; 7 Department of Internal Medicine, College of Medicine, National Taiwan University, Taipei, 100 Taiwan; 8 YongLin Institute of Health, National Taiwan University, Taipei, Taiwan; 9 Institute of Oral Medicine, College of Medicine, National Cheng Kung University, Tainan, Taiwan

**Keywords:** Lnc-MLETA1, Lung cancer metastasis, Exosome, miR-186-5p, miR-497-5p, IGF1R, EGFR

## Abstract

**Background:**

Lung cancer is the most common and deadliest cancer worldwide, and approximately 90% of all lung cancer deaths are caused by tumor metastasis. Tumor-derived exosomes could potentially promote tumor metastasis through the delivery of metastasis-related molecules. However, the function and underlying mechanism of exosomal long noncoding RNA (lncRNA) in lung cancer metastasis remain largely unclear.

**Methods:**

Cell exosomes were purified from conditioned media by differential ultracentrifugation and observed using transmission electron microscopy, and the size distributions were determined by nanoparticle tracking analysis. Exosomal lncRNA sequencing (lncRNA-seq) was used to identify long noncoding RNAs. Cell migration and invasion were determined by wound-healing assays, two-chamber transwell invasion assays and cell mobility tracking. Mice orthotopically and subcutaneously xenografted with human cancer cells were used to evaluate tumor metastasis in vivo. Western blot, qRT‒PCR, RNA-seq, and dual-luciferase reporter assays were performed to investigate the potential mechanism. The level of exosomal lncRNA in plasma was examined by qRT‒PCR. MS2-tagged RNA affinity purification (MS2-TRAP) assays were performed to verify lncRNA-bound miRNAs.

**Results:**

Exosomes derived from highly metastatic lung cancer cells promoted the migration and invasion of lung cancer cells with low metastatic potential. Using lncRNA-seq, we found that a novel lncRNA, lnc-MLETA1, was upregulated in highly metastatic cells and their secreted exosomes. Overexpression of lnc-MLETA1 augmented cell migration and invasion of lung cancer. Conversely, knockdown of lnc-MLETA1 attenuated the motility and metastasis of lung cancer cells. Interestingly, exosome-transmitted lnc-MLETA1 promoted cell motility and metastasis of lung cancer. Reciprocally, targeting lnc-MLETA1 with an LNA suppressed exosome-induced lung cancer cell motility. Mechanistically, lnc-MLETA1 regulated the expression of EGFR and IGF1R by sponging miR-186-5p and miR-497-5p to facilitate cell motility. The clinical datasets revealed that lnc-MLETA1 is upregulated in tumor tissues and predicts survival in lung cancer patients. Importantly, the levels of exosomal lnc-MLETA1 in plasma were positively correlated with metastasis in lung cancer patients.

**Conclusions:**

This study identifies lnc-MLETA1 as a critical exosomal lncRNA that mediates crosstalk in lung cancer cells to promote cancer metastasis and may serve as a prognostic biomarker and potential therapeutic target for lung cancer diagnosis and treatment.

**Supplementary Information:**

The online version contains supplementary material available at 10.1186/s13046-023-02859-y.

## Background

Lung cancer is the most common and deadliest cancer worldwide [1]. Non-small cell lung cancer (NSCLC) accounts for approximately 85% of all lung cancers [2]. NSCLC patients have no typical clinical symptoms in the early stages; thus, most patients are diagnosed with advanced-stage disease at the time of their initial diagnosis, and the five-year survival rate is less than 10% [3, 4]. In addition, approximately 90% of all lung cancer-related deaths are due to metastasis. Therefore, elucidation of the underlying mechanisms of lung cancer metastasis and identification of early biomarkers for lung cancer diagnostics and treatment are urgently needed.

Exosomes are extracellular membrane nanovesicles (30–150 nm) that can be secreted by most types of cells, including immune cells, fibroblasts, adipocytes, epithelial cells, and especially tumor cells [5]. Emerging evidence indicates that exosomes can potentially facilitate cell motility and tumor metastasis through the transfer of protein and noncoding RNA [6]. For instance, Shang et al. found that exosome-delivered circPACRGL promotes colorectal cancer cell migration and invasion through the miR-142-3p/miR-506-3p-TGF-β1 axis [7]. Wang et al. found that exosome-derived GIPC2 facilitates prostate cancer metastasis via the activation of WNT-β-catenin cascades [8]. Qiu et al. found that exosome-transmitted miR-519a-3p enhances liver metastasis by targeting DUSP2 and activating the MAPK/ERK pathway [9]. These studies suggest that exosomes play an important role in intercellular communication and are involved in tumor progression and metastasis.

Long noncoding RNAs (lncRNAs) are a class of transcripts that are longer than 200 nucleotides and lack protein-coding ability [10]. Accumulating evidence has shown that lncRNAs are involved in multiple regulatory mechanisms of gene expression and modulate many physiological and pathological functions, such as metabolism, proliferation, apoptosis, drug resistance, and especially tumor metastasis [11–16]. Recent studies have suggested that the molecular mechanisms of lncRNAs depend on lncRNA cellular localization [17]. In the cytoplasm, lncRNAs participate in post-transcriptional modification by acting as competing endogenous RNAs to decoy microRNAs and increase the expression of downstream targets. Moreover, lncRNAs can enhance protein stability by directly interacting with the target protein and influence mRNA translation by complementarily binding to mRNAs. In the nucleus, lncRNAs can be involved in transcriptional regulation by recruiting transcription factors or modifying transcription factor activity. Recently, many studies have revealed that lncRNAs modulate tumor metastasis in various cancers [18]. For example, Li et al. found that lncRNA UBE2CP3 promotes gastric cancer metastasis by regulating the miR-138-5p/ITGA2 axis [19]. Wu et al. found that LINC00941 facilitates colorectal cancer metastasis by directly binding the SMAD4 protein and competing with ubiquitin ligase to prevent SMAD4 protein degradation [20]. Wen et al. found that lncRNA NEAT1 promotes lung and bone metastasis of prostate cancer by modulating Pol II ser2 phosphorylation [21]. Collectively, these studies suggest that lncRNAs play a critical role in tumor metastasis. In addition, some studies have shown that exosomal lncRNAs participate in lung cancer aggressiveness and anticancer therapy resistance [22–25]. However, the effect and mechanism of exosomal lncRNAs on lung cancer metastasis is still unknown.

In this study, we found a novel lncRNA, ENST00000563763, that was upregulated in highly metastatic lung cancer cells and their secreted exosomes. Therefore, we named this lncRNA metastatic lung cancer cell-derived exosome transmitted lncRNA 1 (MLETA1). Functional assays indicated that lnc-MLETA1 promoted cell motility and metastasis in lung cancer. Interestingly, exosome-transmitted lnc-MLETA1 facilitated cell motility and metastasis of lung cancer. Mechanistically, lnc-MLETA1 regulated the expression of EGFR and IGF1R and promoted cell motility by sponging miR-186-5p and miR-497-5p. Our findings suggest that lnc-MLETA1 plays a key role in lung cancer metastasis and may serve as a novel biomarker and potential therapeutic target for lung cancer diagnostics and treatment.

## Methods

### Cell culture

The human lung adenocarcinoma cell line CL1-0 and CL1-5 cells were cultured in RPMI 1640 medium supplemented with 10% fetal bovine serum (FBS, Gibco). The stable cells were selected in RPMI 1640 medium supplemented with 10% FBS and 800 μg/ml G418. All cells were cultured in a humidified atmosphere containing 5% CO_2_ at 37 ˚C.

### Conditioned media harvest

CL1-0 (1 × 10^6^ cells) and CL1-5 (1 × 10^6^ cells) were seeded in 100-mm dishes. After 24 h, the cells were washed with phosphate buffered saline (PBS) two times and were incubated in RPMI-1640 medium containing exosome-depleted FBS for 48 h. After incubation, the conditioned media were harvested and centrifuged at 300 g for 15 min and at 2,000 g for 15 min at 4 ˚C to remove cells.

### Purification of exosomes by differential ultracentrifugation

CL1-0 (1 × 10^6^ cells) and CL1-5 (1 × 10^6^ cells) were seeded in 100-mm dishes. After 24 h, the cells were washed with PBS two times and incubated in RPMI-1640 medium containing exosome-depleted FBS for 48 h. After incubation, exosomes were purified from the conditioned media by differential ultracentrifugation. Briefly, conditioned media were centrifuged at 300 g for 15 min and at 2,000 g for 15 min at 4 ˚C to remove cells. And then supernatant was centrifuged at 10,000 g for 45 min at 4 ˚C to remove cell debris. Finally, exosomes were pelleted by ultracentrifugation at 100,000 g for 70 min at 4 ˚C. Exosomes were resuspended in PBS and collected by ultracentrifugation again at 100,000 g for 70 min.

### Transmission electron microscopy

10 μl of exosomes were dropped onto Formvar-carbon-coated 200-mesh copper electron microscopy grids, incubated for 5 min at room temperature (RT) and the excess solution were removed by filter paper. After stained by 2% uranyl acetate for 1 min at RT, the grids were washed with ddH_2_O twice. The samples were observed using transmission electron microscopy.

### Nanoparticle tracking analysis

Analysis of absolute size distribution and concentration of exosomes were determined using Nanoparticle tracking analysis. Exosomes were diluted in 1 ml PBS and mixed well, then the diluted exosomes were injected into the NanoSight NS300 instrument, and particles were automatically tracked and sized based on Brownian motion and the diffusion coefficient. Filtered PBS was used as controls.

### Western blot analysis

Cells were washed with PBS two times and lysed in RIPA lysis buffer containing 0.25% Sodium deoxycholate, 0.1% SDS a, 1% Triton X-100 in 1 × PBS, 1 mM Na_3_VO_4_, 10 mM NaF, and protease inhibitor (Roche), for 10 min on ice. After collecting the cell lysates by scraping, centrifuged the lysates at 12,000 rpm for 30 min at 4 °C. Collected clear supernatants and measured protein concentration using a Bradford protein assay (Bio-Rad). The cell lysates (20 μg) and exosomes (10 μl) were loaded and proteins were separated by 10% sodium dodecyl sulfate polyacrylamide gel electrophoresis (SDS-PAGE). Next, the protein was transferred to PVDF membrane (0.45 μm, Millipore) in transfer buffer at 400 mA for 1 h and blocked in 0.1% TBST with 5% non-fat milk for 1 h. Subsequently, the membrane was probed with primary antibodies in 0.1% TBST with 5% BSA at 4 °C overnight. Then blots were incubated with the appropriate horseradish peroxidase-conjugated (HRP-conjugated) secondary antibodies (Jackson ImmunoResearch) in 0.1% TBST with 5% non-fat milk at RT for 1 h, and the bound antibodies were visualized using ECL staining (PerkinElmer).

### Antibodies

Primary antibodies used for immunoblotting were listed below: anti-CD9 (D8O1A; Cell Signaling), anti-TSG101 (GTX118736; GeneTex), anti-Flotillin-1 (GTX104769; GeneTex), anti-Calnexin (GTX109669; GeneTex), anti-GFP (sc-9996; Santa Cruz), anti-Ago2 (ab186733; Abcam), anti-GST (M0006; AbOmics), anti-EGFR (#2232; Cell Signaling), anti-IGF1R (D23H3; Cell Signaling), anti-β-Actin (AC-15; Sigma-Aldrich).

### In vitro wound-healing assay

In vitro wound-healing assays were performed with Ibidi Culture-Inserts (Blossom Biotechnologies). CL1-0 (1.8 × 10^4^ cells) and CL1-5 (3 × 10^4^ cells) were seeded in each insert for 24 h, and the inserts were removed. Photographs were taken at 0, 10, 16 and 24 h in the wound gap with 40 × magnification. Finally, the number of migrated cells was counted.

### Transwell invasion assay

Transwell invasion assays were performed with 24-well polycarbonate Transwell filters (pore size 8 μm, Costar 3422) coated with 30 μg of Matrigel (BD Biosciences, NY, USA). CL1-0 (1.8 × 10^4^ cells) and CL1-5 (3 × 10^4^ cells) were seeded in the upper chamber containing serum-free medium, and RPMI-1640 medium containing 10% FBS were added in the lower chamber for 24 h and 16 h, respectively. On the upper surface of the filter, non-penetrating cells were removed with a cotton swab. Penetrating cells were stained by Liu’s stain (ASK). Finally, the number of invaded cells was counted at 40 × magnification in five different fields per filter.

### RNA-sequencing analysis

Total RNA was fragmented through heat treatment and then reverse transcribed into cDNA using random primers and reverse transcriptase. Library construction was completed by performing end trimming, adding adapters, PCR amplification, and magnetic bead purification. Quality-verified libraries were sequenced on the Illumina NovaSeq 6000 platform with a specification of 150 PE sequencing. The raw data was processed using fastp to eliminate low-quality bases, adapters, and reads containing excessive unknown bases (N), resulting in clean reads. After removing ribosomal RNA, the reads depleted of rRNA were aligned to the reference genome. Differential gene expression analysis was conducted and identified using edgeR.

### Cell proliferation assay

lnc-MLETA1-knockdown and control CL1-5 cells (3 × 10^3^ cells per well) were seeded in 100 μl medium in a 96-well plate. Proliferation was measured every 24 h. After removing the culture medium, 90 μl medium mixed with 10 μl WST-1 reagent (Roche) were added to each well. After 40 min incubation, cell viability was determined by measuring the absorbance at 450 nm using a microplate reader.

### Soft-agar colony formation assay

lnc-MLETA1-knockdown or control CL1-5 cells (3 × 10^3^ cells per well) in RPMI medium containing 0.35% agarose gel were seeded into 6-well plates containing 0.7% agarose gel in the lower layer. The plates were incubated at 37 ˚C with 5% CO_2_ for 2 weeks to facilitate foci formation. Subsequently, the colonies were stained with 0.1% crystal violet and counted under a microscope.

### Cellular RNA isolation

The cells in 60-mm dish were lysed with 1 ml TRIzol reagent (Invitrogen) at RT for 10 min. Then the lysate was added 200 μl of chloroform. The mixture was incubated at RT for 3 min and then centrifuged at 12,000 rpm for 20 min at 4 ˚C. The upper supernatant was transferred into fresh tube and added 500 μl isopropanol. The mixture was incubated at RT for 10 min and then centrifuged at 12,000 rpm for 20 min at 4 ˚C. Then upper supernatant was completely removed. The RNA pellet was washed in 1 ml cold 75% ethanol in DEPC-ddH_2_O and centrifuged at 12,000 rpm for 10 min at 4 ˚C. Finally, the RNA pellet was air-dried for 10 min and then dissolved in 20 μl DEPC-ddH_2_O.

### Exosomal RNA isolation

Total RNA from exosomes were extracted by the Direct-zol™ RNA kit (Zymo Research) following the manufacturer's instructions.

### Quantitative real-time polymerase chain reaction (qRT-PCR)

The purity and concentration of isolated RNA were determined by Nanodrop. 1 μg of cellular total RNA or 20 ng of exosomal total RNA were reverse-transcribed to cDNA using the Random Hexamers by SuperScript III Reverse Transcriptase (Invitrogen). Quantitative RT-PCR analysis were performed using the SYBR Green PCR Master Mix (Applied Biosystems). GAPDH, 18S rRNA and U6 snRNA were used for normalization.

### Plasmid construction

The cDNA of lncRNA was amplified by AccuPrime Pfx DNA Polymerase (Invitrogen) and subcloned into the pcDNA3.1 ( +) vector.

### Transfection

CL1-0 (4 × 10^5^ cells) and CL1-5 (4 × 10^5^ cells) were seeded in 60-mm dishes. After 24 h, plasmids and locked nucleic acid (LNA™) were transfected into the cells using Lipofectamine™ 2000 transfection reagents (Invitrogen) and Lipofectamine™ RNAiMAX transfection reagent (Invitrogen), respectively. 12.5 μl lipofectamine 2000 or 18 μl lipofectamine RNAiMAX was mixed with 600 μl OPTI-MEM medium (Gibco) and 5 μg plasmids or 6 μl locked nucleic acid was mixed with another 600 μl OPTI-MEM medium. After incubation at RT for 5 min, the two mixtures were mixed together. After incubation at RT for 20 min, the culture medium was removed, and the cells were washed with PBS. Then the mixture was added into cells and cells were incubated at 37 in 5% CO_2_. After 4–6 h, the culture medium was replaced with fresh medium. Post 48-h post-transfection, functional assay was performed on the cells.

### MS2-tagged RNA affinity purification

MS2-tagged RNA affinity purification was performed as previously described [26]. Briefly, CL1-0 cells were co-transfected with either 6 μg of pMS2-lnc-MLETA1 or pMS2 along with 2 μg of pMS2-GST. Following 48 h of transfection, the cells were lysed in 1000 μl of lysis buffer containing 20 mM Tris–HCl at pH 7.5, 100 mM KCl, 5 mM MgCl_2_, 0.5% NP-40, 10 mM dithiothreitol (DTT), protease inhibitors, and RNase inhibitor. The supernatant was incubated with a slurry of 50 μl glutathione (GSH) agarose beads overnight at 4 °C, followed by washing with NT2 buffer containing 50 mM Tris–HCl at pH 7.5, 150 mM NaCl, 1 mM MgCl_2_, and 0.05% NP-40. Western blot analysis was performed using primary antibodies against Ago2 and glutathione S-transferase (GST). For the qRT‒PCR assay, the supernatant was incubated with 0.1% SDS and 0.5 mg/ml Proteinase K for RNA purification before validating the expression levels of lncRNAs and miRNAs.

### Xenograft animal model

For orthotopic xenograft model, 1 × 10^5^ CL1-5-GL or lnc-MLETA1-knockdown cells were suspended in 10 μl PBS and mixed with 10 μl Matrigel and intrapulmonary injection transplanted into the right lung of 6 ~ 8-week-old male nonobese diabetic/severe combined immunodeficiency (NOD-SCID) mice. After inoculation, tumor cells metastasizing from the right lung to the left lung were monitored and evaluated by the IVIS spectrum in vivo imaging system (PerkinElmer) every week for 4 weeks. After the mice were sacrificed, the whole lung was excised, formalin-fixed, paraffin-embedded, and subjected to hematoxylin–eosin staining. The number and size of tumor metastasis in the lung were observed and counted under a microscope. For subcutaneous xenograft model, 1 × 10^6^ CL1-0-GL cells were suspended in 100 μl PBS and mixed with 100 μl Matrigel and subcutaneously injection transplanted into the NOD-SCID mice. After inoculation, NOD-SCID mice were injected intratumorally with 20 μg exosomes derived from lnc-MLETA1-overexpressing or control CL1-0 cells twice a week and the lung metastasis were monitored and evaluated by IVIS spectrum in vivo imaging system for 6 weeks. After the mice were sacrificed, the whole lung was excised, formalin-fixed, paraffin-embedded, and subjected to hematoxylin–eosin staining. The number and size of tumor metastasis in the lung were observed and counted under a microscope. The animal study protocol was approved by the Institutional Animal Care and Use Committee of National Cheng Kung University (IACUC Approval No. 107119, Tainan, Taiwan).

### Statistical analysis

All statistical analyses were performed using GraphPad Prism 8.0. Results are presented as mean ± SD. A *P*-value < 0.05 was considered significant. The survival rate data of lung cancer patients were downloaded from the KM Plotter Online Tool (http://www.kmplot.com) and were analyzed by Kaplan–Meier analysis with the log-rank test. The correlation of exosomal lnc-MLETA1 levels isolated from plasma samples of lung cancer patients with clinical characteristics was analyzed using the Chi-squared test.

## Results

### Exosomes derived from highly metastatic lung cancer cells promote cell migration and invasion of poorly metastatic lung cancer cells.

**Fig. 1:**
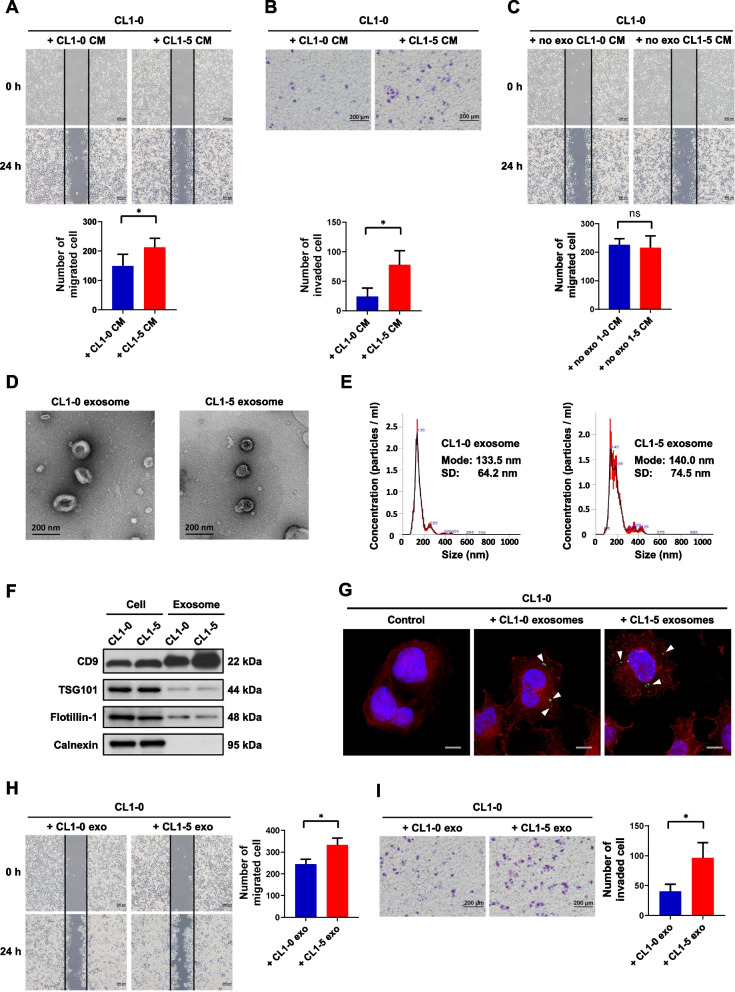
Exosomes derived from high metastatic lung cancer cells promotes cell migration and invasion of low metastatic lung cancer cells. **A** Upper: representative images of wound-healing assay of CL1-0 cells pre-incubated with CL1-0 or CL1-5 conditioned media for 48 h. Scale bar, 200 μm. Lower: the number of migrated cells was calculated. **B** Upper: representative images of transwell invasion assay of CL1-0 cells pre-incubated with CL1-0 or CL1-5 conditioned media for 48 h. Scale bar, 200 μm. Lower: the number of invaded cells was calculated. **C** Upper: representative images of wound-healing assay of CL1-0 cells pre-incubated with CL1-0 or CL1-5 exosome-depleted conditioned media for 48 h. Scale bar, 200 μm. Lower: the number of migrated cells was calculated. **D** Representative transmission electron microscopy images of exosomes derived from CL1-0 and CL1-5 cells. Scale bar, 200 nm. **E** Nanoparticle tracking analysis of the size distributions and number of exosomes derived from CL1-0 and CL1-5 cells. **F** Western blot analysis of exosome marker CD9, TSG101 and Flotillin-1 in CL1-0 and CL1-5 cells and exosomes. Calnexin was used as the negative controls. **G** Immunofluorescence assay of GFP in CL1-0 cells pre-incubated with CD9-GFP containing exosomes derived from CL1-0 or CL1-5 cells. Cells were stained with rhodamine phalloidin and DAPI for F-actin and nuclei, respectively. White arrowheads indicate the CD9-GFP containing exosomes. Scale bar, 20 μm. **H** Left: representative images of wound-healing assay of CL1-0 cells pre-incubated with CL1-0 or CL1-5 exosomes for 48 h. Scale bar, 200 μm. Right: the number of migrated cells was counted. **I** Left: representative images of transwell invasion assay of CL1-0 cells pre-incubated with CL1-0 or CL1-5 exosomes for 48 h. Scale bar, 200 μm. Right: the number of invaded cells was counted. Results are presented as mean ± SD from three independent experiments. *P < 0.05. Two-tailed Student’s *t*-test

To determine whether exosomes derived from highly metastatic CL1-5 lung cancer cells could enhance the migratory and invasive abilities of poorly metastatic CL1-0 lung cancer cells, we incubated CL1-0 cells with conditioned media from CL1-0 and CL1-5 cells. Wound-healing assays and transwell invasion assays showed that conditioned media from CL1-5 cells significantly increased the migration and invasion of CL1-0 cells compared with CL1-0 conditioned media (Fig. 1A and B). To further determine whether the effect of conditioned media on cell motility is mediated by exosomes, we performed a wound-healing assay on CL1-0 cells incubated with exosome-depleted conditioned media from CL1-0 and CL1-5 cells. The results showed that the exosome-depleted conditioned medium of CL1-5 cells had no significant influence on CL1-0 cell migration (Fig. 1C). These data suggested that the increased migratory and invasive abilities of CL1-0 cells are primarily due to the transfer of exosomes from CL1-5 cells in the extracellular microenvironment.

To investigate the effect of exosomes derived from CL1-5 cells on the migratory and invasive abilities of CL1-0 cells, we isolated exosomes from the conditioned media of CL1-0 and CL1-5 cells by ultracentrifugation. The exosomes were characterized by transmission electron microscopy, western blot analysis and nanoparticle tracking analysis. Transmission electron microscopy of negatively stained exosomes revealed cup-shaped membrane vesicles (Fig. 1D). The nanoparticle tracking analysis indicated that the sizes of the exosomes derived from CL1-0 and CL1-5 cells were 133.5 ± 64.2 nm and 140.0 ± 74.5 nm, respectively (Fig. 1E). Western blot analysis showed the presence of the exosomal markers CD9, TSG101, and Flotillin-1 but not the endoplasmic reticulum protein calnexin in the exosomal samples (Fig. 1F). These data demonstrated that the vesicles purified from CL1-0 and CL1-5 cells were exosomes. To further determine whether exosomes can be directly transferred between cells, we isolated exosomes from the conditioned media of cells transiently transfected with the pEGFP-N-CD9 plasmid. Western blot analysis revealed that GFP was expressed in exosomes from the GFP-tagged CD9-overexpressing CL1-0 and CL1-5 cells (Supplementary Fig. S1A). Next, we incubated CL1-0 cells with CD9-GFP-containing exosomes for 8 h and observed GFP expression in the treated CL1-0 cells by confocal microscopy. The results showed that green fluorescence signals were detected in the treated CL1-0 cells but not in the untreated CL1-0 cells (Fig. 1G). These data indicated that exosomes derived from CL1-0 and CL1-5 cells can be taken up by CL1-0 cells. To determine whether exosome transfer from CL1-5 cells to CL1-0 cells can promote cell migration and invasion, we incubated CL1-0 cells with exosomes derived from CL1-0 and CL1-5 cells. Wound-healing assays and transwell invasion assays showed that exosomes derived from CL1-5 significantly increased the migration and invasion of CL1-0 cells compared with CL1-0 exosomes (Fig. 1H and I). Taken together, these data suggested that the exosomes derived from CL1-5 cells facilitate cell migration and invasion of CL1-0 cells.

### lnc-MLETA1 promotes the migration and invasion of lung cancer cells in vitro

**Fig. 2:**
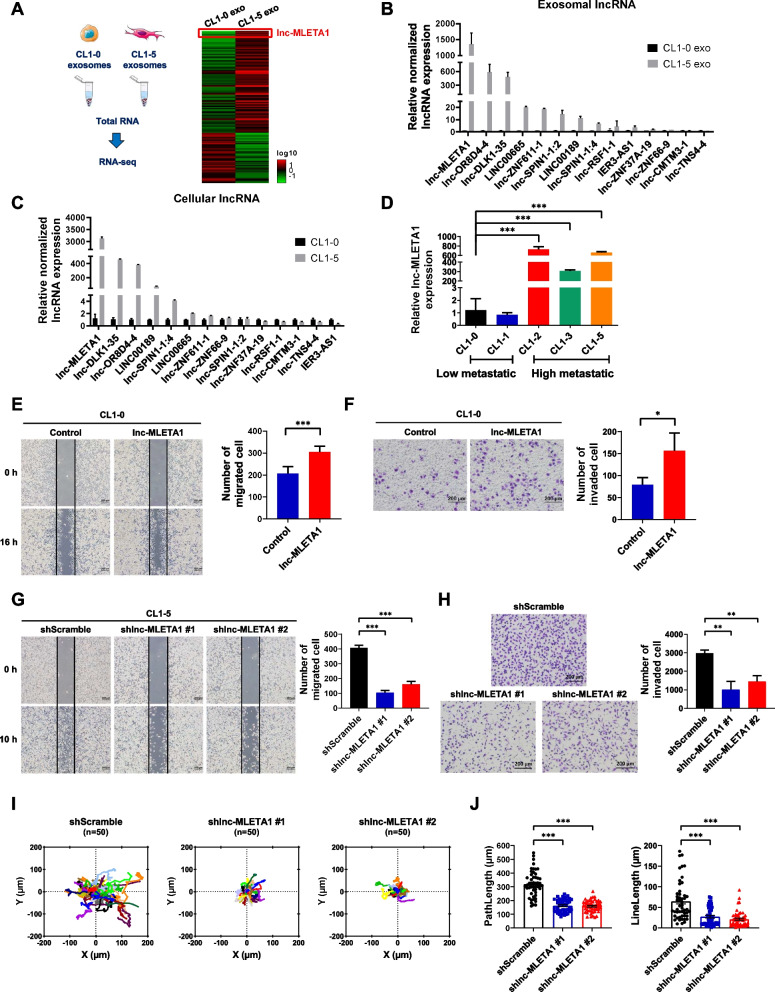
lnc-MLETA1 promotes migration and invasion of lung cancer cells in vitro. **A** RNA-sequencing of total RNA extracted from CL1-0 exosomes and CL1-5 exosomes are presented in a heatmap. **B** and **C** qRT-PCR analysis of lnc-MLETA1 and other candidate lncRNA expression in CL1-0 and CL1-5 exosomes (**B**) and cells (**C**). **D** qRT-PCR analysis of lnc-MLETA1 expression between low metastatic cells and high metastatic lung cancer cells. **E** Left: representative images of wound-healing assay of lnc-MLETA1-overexpressing and control CL1-0 cells for 16 h. Scale bar, 200 μm. Right: the number of migrated cells was counted. **F** Left: representative images of transwell invasion assay of lnc-MLETA1-overexpressing and control CL1-0 cells for 24 h. Scale bar, 200 μm. Right: the number of invaded cells was counted. **G** Left: representative images of wound-healing assay of lnc-MLETA1-knockdown and control CL1-5 cells for 10 h. Scale bar, 200 μm. Right: the number of migrated cells was counted. **H** Left: representative images of transwell invasion assay of lnc-MLETA1-knockdown and control CL1-5 cells for 16 h. Scale bar, 200 μm. Right: the number of invaded cells was counted **I** The XY position plots of single-cell tracking analysis of lnc-MLETA1-knockdown and control CL1-5 cells. **J** The path length and line length of cell migration of lnc-MLETA1-knockdown and control CL1-5 cells. Results are presented as mean ± SD from three independent experiments. *P < 0.05, **P < 0.01, ***P < 0.001. Two-tailed Student’s *t*-test

Recently, many studies have shown that exosomal miRNAs and proteins promote tumor metastasis [27–30]. However, the effect of exosomal lncRNAs on cell motility and tumor metastasis is still unknown. Therefore, we sought to explore whether exosomal lncRNAs can alter the motility of lung cancer cells. To identify lncRNAs in exosomes that may play roles in cell motility, we performed lncRNA sequencing on CL1-0 and CL1-5 exosomes. The heatmap showed the top 100 most increased and the top 50 most decreased lncRNAs among 489 differentially-expressed lncRNAs (fold change > 5). There are 309 upregulated lncRNAs and 180 downregulated lncRNAs in CL1-5 exosomes compared with CL1-0 exosomes (Fig. 2A). The results showed that a novel lncRNA, ENST00000563763, was upregulated in CL1-5 exosomes compared with CL1-0 exosomes. We named it lnc-MLETA1 (metastatic lung cancer cell-derived exosome transmitted lncRNA 1). Next, we used qRT‒PCR analysis to confirm the expression of several lncRNAs in CL1-0 and CL1-5 cells and their exosomes. The results showed that the expression of lnc-MLETA1 was highest in CL1-5 cells and exosomes compared with that of CL1-0 cells and exosomes (Fig. 2B and C). Moreover, we compared the expression of lnc-MLETA1 in poorly and highly metastatic lung cancer cells by qRT‒PCR analysis and found that the expression of lnc-MLETA1 was significantly upregulated in highly metastatic lung cancer cells (Fig. 2D), suggesting that lnc-MLETA1 may be associated with the migratory and invasive abilities. Furthermore, the bioinformatic analysis showed that the sequence of lnc-MLETA1 was identified without protein-coding potential by Coding Potential Assessment Tool (CPAT) (Supplementary Fig. S1B). The secondary structure of lnc-MLETA1 was predicted by RNAfold web server (Supplementary Fig. S1C). lnc-MLETA1 is located on chromosome 17, near WSCD1 (Supplementary Fig. S1D). The ChIP-seq dataset GSE225332 indicated the enrichment of active histone markers, specifically H3K27ac and H3K4me3, within the genomic regions encompassing lnc-MLETA1 in lung cancer cells, suggesting a more open and accessible chromatin structure (Supplementary Fig. S1E). In addition, we utilized the KM Plotter Online Tool to analyze the correlation between the expression of lnc-MLETA1 and the survival rates of cancer patients, including AML, breast cancer, colon cancer, gastric cancer, myeloma, and ovarian cancer. The results showed that the expression of lnc-MLETA1 was not significantly correlated with patients' survival in AML, breast cancer, colon cancer, gastric cancer, myeloma, and ovarian cancer (Supplementary Fig. S1F-K). It seems that lnc-MLETA1 is specific and important for lung cancer.

To further determine the role of lnc-MLETA1 in cell motility, we overexpressed lnc-MLETA1 in CL1-0 cells by transfection of lnc-MLETA1 plasmids (Supplementary Fig. S2A). Wound-healing assays and transwell invasion assays showed that overexpression of lnc-MLETA1 significantly increased the migration and invasion of CL1-0 cells compared with those of the control group (Fig. 2E and F). Conversely, we silenced lnc-MLETA1 in CL1-5 cells by lentivirus-based short hairpin RNAs (shRNAs) (Supplementary Fig. S2B). Wound-healing assays and transwell invasion assays showed that the knockdown of lnc-MLETA1 significantly decreased the migration and invasion of CL1-5 cells compared with the controls (Fig. 2G and H). Moreover, single-cell tracking analysis revealed that knockdown of lnc-MLETA1 significantly decreased the path length and line length of CL1-5 cell migration compared with those in the control group (Fig. 2I and J). Furthermore, the cell proliferation assay and soft agar colony formation assay indicated that knockdown of lnc-MLETA1 significantly decreased the cell growth and anchorage-independent growth of CL1-5 cells compared with the controls (Supplementary Fig. S2C and D). Collectively, these data suggested that lnc-MLETA1 plays a critical role in cell motility and growth.

### Exosomal lnc-MLETA1 promotes lung cancer cell migration and invasion in vitro

**Fig. 3:**
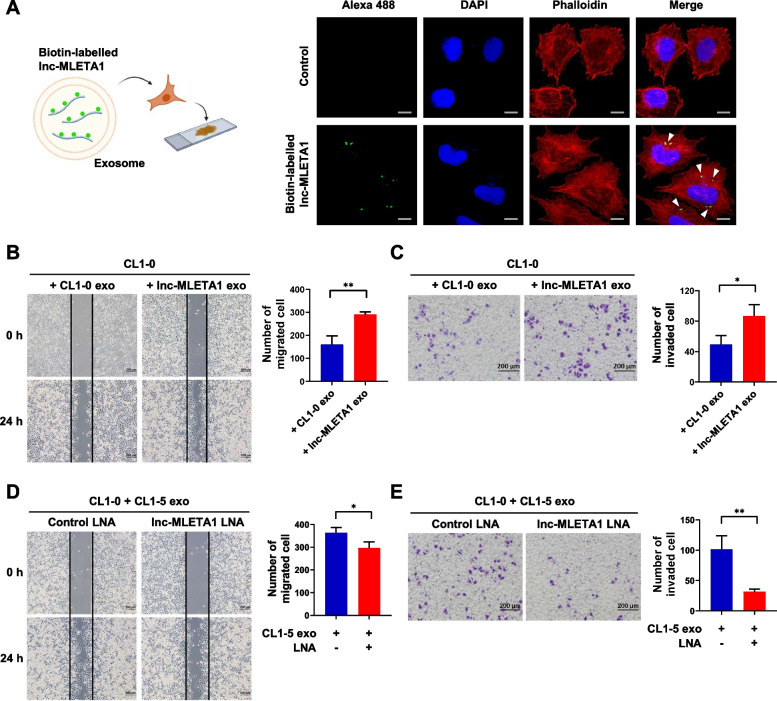
Exosomal lnc-MLETA1 promotes lung cancer cell migration and invasion in vitro. **A** Immunofluorescence assay of biotin-labelled lnc-MLETA1 in CL1-0 cells pre-incubated with exosomes derived from biotin-labelled lnc-MLETA1-transfected cells or control CL1-0 cells. Cells were stained with rhodamine phalloidin and DAPI for F-actin and nuclei, respectively. White arrowheads indicate the biotin-labelled lnc-MLETA1. Scale bar, 20 μm. **B** Left: representative images of wound-healing assay of CL1-0 cells pre-incubated with exosomes derived from lnc-MLETA1-overexpressing or control CL1-0 cells for 48 h. Scale bar, 200 μm. Right: the number of migrated cells was counted. **C** Left: representative images of transwell invasion assay of CL1-0 cells pre-incubated with exosomes derived from lnc-MLETA1-overexpressing or control CL1-0 cells for 48 h. Scale bar, 200 μm. Right: the number of invaded cells was counted. **D** Left: representative images of wound-healing assay of CL1-0 cells pre-incubated with CL1-5 exosomes and transfected with lnc-MLETA1 LNA or control LNA for 48 h. Scale bar, 200 μm. Right: the number of migrated cells was counted. **E** Left: representative images of transwell invasion assay of CL1-0 cells pre-incubated with CL1-5 exosomes and transfected with lnc-MLETA1 LNA or control LNA for 48 h. Scale bar, 200 μm. Right: the number of invaded cells was counted. Results are presented as mean ± SD from three independent experiments. *P < 0.05, **P < 0.01. Two-tailed Student’s *t*-test

To observe the exosome-mediated transmission of lnc-MLETA1, we biotinylated lnc-MLETA1 and transfected it into CL1-0 cells to collect the exosomes. Subsequently, the purified exosomes were added to CL1-0 cells. Confocal microscopy analysis showed that CL1-0 cells incubated with exosomes containing biotinylated lnc-MLETA1 had obvious green fluorescent signals, but control cells did not (Fig. 3A). To further determine whether the exosome-mediated transfer of lnc-MLETA1 promotes cell migration and invasion, we first isolated exosomes from lnc-MLETA1-overexpressing CL1-0 cells. qRT‒PCR analysis showed that the levels of lnc-MLETA1 were significantly increased in the lnc-MLETA1-overexpressing CL1-0 exosomes compared with the CL1-0 exosomes (Supplementary Fig. S3A). The levels of lnc-MLETA1 in the CL1-0 cells treated with lnc-MLETA1-overexpressing exosomes were significantly higher than those in the cells treated with control exosomes (Supplementary Fig. S3B). Wound-healing assays and transwell invasion assays showed that the lnc-MLETA1-overexpressing exosomes significantly increased the migration and invasion of CL1-0 cells compared with the control exosomes (Fig. 3B and C). These data indicated that exosome-mediated transfer of lnc-MLETA1 could promote cell migration and invasion.

To determine whether the effect of CL1-5 exosomes on cell motility is mediated by the delivery of lnc-MLETA1, we first detected the levels of lnc-MLETA1 in the CL1-0 cells treated with CL1-0 or CL1-5 exosomes. The results showed that lnc-MLETA1 was significantly increased in the CL1-0 cells treated with CL1-5 exosomes compared with those treated with CL1-0 exosomes (Supplementary Fig. S3C). We incubated CL1-0 cells with CL1-5 exosomes and transfected them with lnc-MLETA1 LNA or control LNA. Wound healing assays and transwell invasion assays showed that knockdown of lnc-MLETA1 significantly inhibited CL1-5 exosome-mediated migration and invasion of CL1-0 cells (Fig. 3D and E). Together, these data demonstrated that exosomes derived from CL1-5 cells promote the migration and invasion of CL1-0 cells by delivering lnc-MLETA1.

### Exosomal lnc-MLETA1 promotes lung cancer metastasis in vivo

**Fig. 4:**
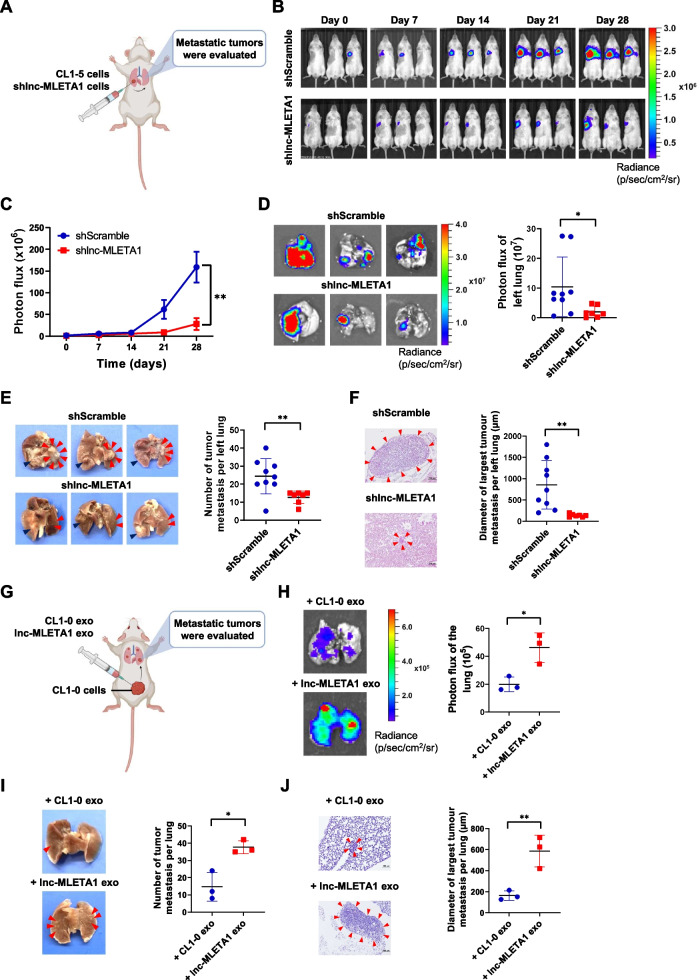
Exosomal lnc-MLETA1 promotes lung cancer metastasis in vivo. **A** The right lungs of NOD-SCID mice were orthotopically xenografted with lnc-MLETA1-knockdown (*n* = 9) or control CL1-5 cells (*n* = 7), and the metastases in the left lungs were observed. **B** Representative bioluminescent images and **C** quantification of bioluminescent imaging signal intensities each week for 4 weeks. **D** Representative bioluminescent images (left) and quantification of bioluminescent imaging signal intensities (right) of the left lung of mice were evaluated by the IVIS system ex vivo. **E** Representative images (left) and the number of metastatic tumors (right) in the left lungs of mice were counted. Deep blue arrow heads indicate primary tumors. Red arrow heads indicate lung metastatic tumors. **F** Representative microscopic images of H&E staining (left) and the diameter of the largest metastatic tumor (right) in the left lungs of mice were counted. Red arrow heads indicate lung metastatic tumors. Scale bar, 100 μm. **G** NOD-SCID mice were subcutaneously xenografted with CL1-0 cells and injected intratumorally with exosomes derived from lnc-MLETA1-overexpressing or control CL1-0 cells twice a week (*n* = 3 mice per group). **H** Representative bioluminescent images (left) and quantification of bioluminescent imaging signal intensities (right) of the lung of mice were evaluated by the IVIS system ex vivo. **I** Representative images (left) and the number of metastatic tumors (right) in the lungs of mice were counted. Red arrow heads indicate lung metastatic tumors. **J** Representative microscopic images of H&E staining (left) and the diameter of the largest metastatic tumor (right) in the lungs of mice were counted. Red arrow heads indicate lung metastatic tumors. Scale bar, 100 μm. Results are presented as mean ± SD. *P < 0.05, **P < 0.01. Two-tailed Student’s *t*-test

To examine the role of lnc-MLETA1 in lung cancer metastasis, we established an orthotopic xenograft model in which luciferase-labeled CL1-5 or lnc-MLETA1-knockdown CL1-5 cells were administered via intrapulmonary injection into the right lungs of 6- to 8-week-old male NOD-SCID mice (Fig. 4A). The IVIS spectrum showed that knockdown of lnc-MLETA1 significantly decreased bioluminescent signals compared with those of the control group (Fig. 4B and C). Moreover, IVIS spectrum ex vivo images showed that knockdown of lnc-MLETA1 significantly reduced the bioluminescent signals in the left lungs compared to the controls (Fig. 4D). Furthermore, knockdown of lnc-MLETA1 significantly decreased the number and size of metastatic lesions in the left lungs (Fig. 4E and F). These data suggested that lnc-MLETA1 is required for lung cancer metastasis.

To further determine whether exosomal lnc-MLETA1 promotes lung cancer metastasis, we subcutaneously xenografted luciferase-labeled CL1-0 cells into NOD-SCID mice and intratumorally injected them with exosomes derived from lnc-MLETA1-overexpressing or control CL1-0 cells twice a week (Fig. 4G). At the primary site, we found that exosomal lnc-MLETA1 augments tumor growth (Supplementary Fig. S4A-E). At the metastatic site, IVIS spectrum ex vivo images showed that exosomal lnc-MLETA1 significantly increased the bioluminescent signals in the lungs compared with the controls (Fig. 4H). Furthermore, exosomal lnc-MLETA1 significantly increased the number and size of lung metastatic lesions (Fig. 4I and J). Collectively, these data suggest that exosomal lnc-MLETA1 promotes lung cancer metastasis.

### lnc-MLETA1 interacts with miR-186-5p and miR-497-5p to promote cell motility

**Fig. 5:**
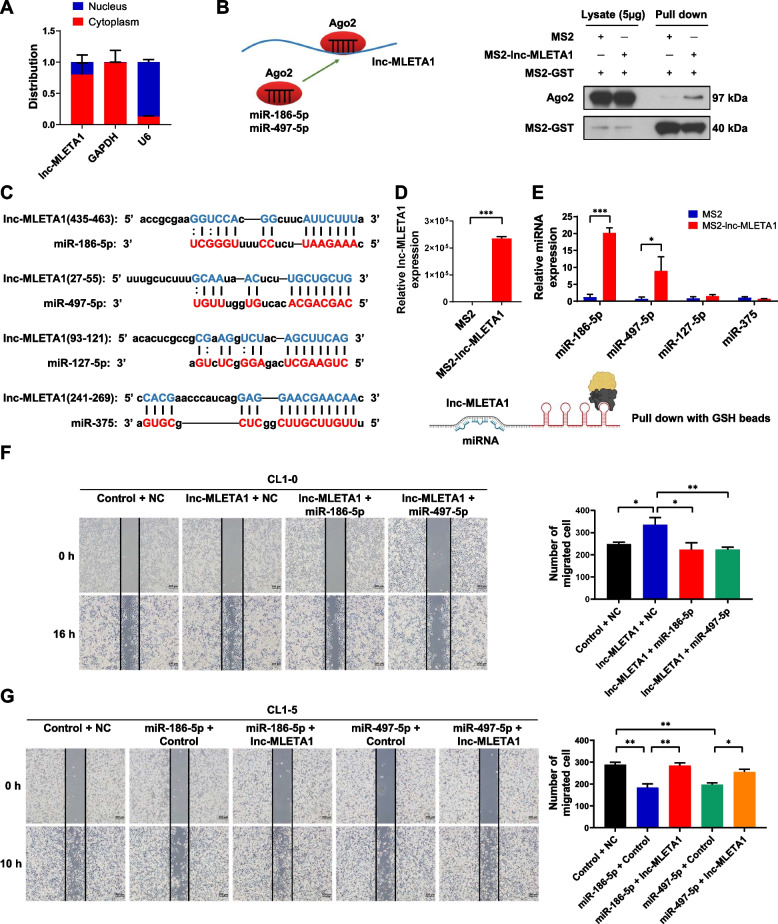
lnc-MLETA1 interacts with miR-186-5p and miR-497-5p to promote cell motility. **A** Cellular RNA fractionation assay of lnc-MLETA1 in CL1-5 cells. **B** Western blot analysis of Ago2 and MS2-GST expression in lysate and the MS2-TRAP RNA pull-down sample. **C** Schematic of predicted binding sites of miR-186-5p, miR-497-5p, miR-127-5p and miR-375 on lnc-MLETA1. **D** qRT-PCR analysis of lnc-MLETA1 expression in the MS2-TRAP RNA pull-down sample. **E** qRT-PCR analysis of miR-186-5p, miR-497-5p, miR-127-5p and miR-375 expression in the MS2-TRAP RNA pull-down sample. **F** Left: representative images of wound-healing assay of CL1-0 cells co-transfected with lnc-MLETA1 plasmids or control plasmids and with miRNA mimics or negative control (NC). Scale bar, 200 μm. Right: the number of migrated cells was counted. **G** Left: representative images of wound-healing assay of CL1-5 cells co-transfected with lnc-MLETA1 plasmids or control plasmids and with miRNA mimics or negative control. Scale bar, 200 μm. Right: the number of migrated cells was counted. Results are presented as mean ± SD from three independent experiments. *P < 0.05, **P < 0.01, ***P < 0.001. Two-tailed Student’s *t*-test

Recently, some studies have shown that lncRNAs can act as miRNA sponges to regulate downstream targets of miRNAs [10]. Therefore, we first determined the distribution of lnc-MLETA1 in CL1-5 cells. Cellular RNA fractionation assays revealed that lnc-MLETA1 is predominantly located in the cytoplasm (Fig. 5A). To investigate whether lnc-MLETA1 can interact with miRNAs, we performed an MS2-tagged RNA affinity purification (MS2-TRAP) assay to verify potential miRNA candidates predicted by LncBase Predicted v.2. The results showed that Ago2 was apparently pulled down by MS2-lnc-MLETA1, indicating that lnc-MLETA1 may interact with miRNA (Fig. 5B). The bioinformatic analysis showed that lnc-MLETA1 contained binding sequences for several miRNAs, including miR-186-5p, miR-497-5p, miR-127-5p and miR-375 (Fig. 5C; Supplementary Fig. S5A). Interestingly, RNA pulldown assays revealed significant increases in miR-186-5p and miR-497-5p in the MS2-lnc-MLETA1 pulldown samples compared to the controls (Fig. 5D and E). These data suggested that lnc-MLETA1 can interact with miR-186-5p and miR-497-5p but not miR-127-5p and miR-375.

To further determine the contribution of lncRNA‒miRNA interactions to cell motility, we performed a wound-healing assay of CL1-0 and CL1-5 cells co-transfected with lnc-MLETA1 and miRNA mimics. The results showed that overexpression of lnc-MLETA1 significantly increased the number of migrated CL1-0 cells, but this effect was abolished by overexpression of miR-186-5p and miR-497-5p (Fig. 5F; Supplementary Fig. S5B-D). Conversely, miR-186-5p and miR-497-5p mimics significantly decreased the migrated cell number in CL1-5 cells, but this effect could be rescued by overexpression of lnc-MLETA1 (Fig. 5G; Supplementary Fig. S5E-G). In addition, lncRNAs and miRNAs did not exhibit any influence on the expression of each other (Supplementary Fig. S5H-J). The levels of miR-186-5p and miR-497-5p did not differ between CL1-0 and CL1-5 cells (Supplementary Fig. S5K and L). Taken together, these data suggest that lnc-MLETA1 promotes cell motility by sponging miR-186-5p and miR-497-5p.

### lnc-MLETA1 promotes cell motility through the miR-186-5p/EGFR and miR-497-5p/IGF1R axes

**Fig. 6:**
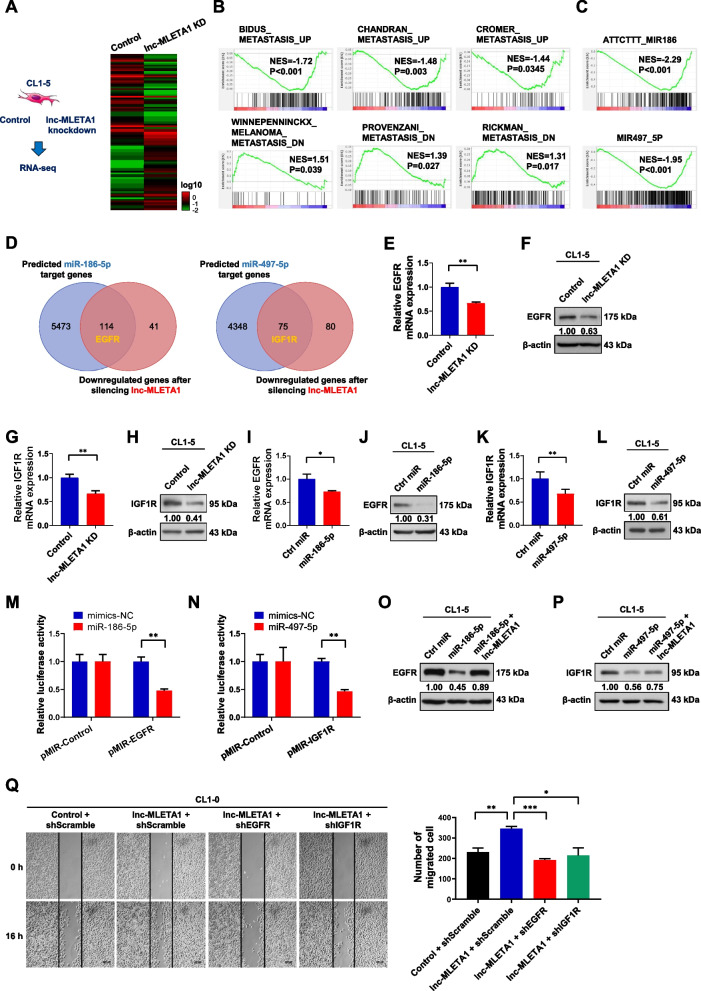
lnc-MLETA1 promotes cell motility through the miR-186-5p-EGFR and miR-497-5p-IGF1R axis. **A** RNA-sequencing of total RNA extracted from lnc-MLETA1-knockdown and control CL1-5 cells are presented in a heatmap. **B** Gene Set Enrichment Analysis (GSEA) of published metastasis gene signatures in lnc-MLETA1-knockdown cells versus control cells. **C** GSEA of miRNA target gene signatures in lnc-MLETA1-knockdown cells versus control cells. **D** Left: venn diagram of intersection of predicted miR-186-5p target gene and downregulated gene after silencing lnc-MLETA1. Right: venn diagram of intersection of predicted miR-497-5p target gene and downregulated gene after silencing lnc-MLETA1. **E** qRT-PCR and **F** Western blot analyses of EGFR expression of lnc-MLETA1-knockdown and control CL1-5 cells. **G** qRT-PCR and **H** Western blot analyses of IGF1R expression of lnc-MLETA1-knockdown and control CL1-5 cells. **I** qRT-PCR and **J** Western blot analyses of EGFR expression of miR-186-5p-overexpressing and control CL1-5 cells. **K** qRT-PCR and **L** Western blot analyses of IGF1R expression of miR-497-5p-overexpressing and control CL1-5 cells. **M** Luciferase activity of CL1-0 cells transfected with miR-186-5p mimics and pMIR-REPORT luciferase plasmid which contain 3'UTR of EGFR. Data are presented as the ratio of Renilla luciferase activity to Firefly luciferase activity. **N** Luciferase activity of CL1-0 cells transfected with miR-497-5p mimics and pMIR-REPORT luciferase plasmid which contain 3'UTR of IGF1R. **O** Western blot analysis of EGFR expression of CL1-5 cells co-transfected with lnc-MLETA1 plasmids or control plasmids and with miR-186-5p mimics or negative control. **P** Western blot analysis of IGF1R expression of CL1-5 cells co-transfected with lnc-MLETA1 plasmids or control plasmids and with miR-497-5p mimics or negative control. **Q** Left: representative images of wound-healing assay of CL1-0 cells co-transfected with lnc-MLETA1 plasmids or control plasmids and with shEGFR, shIGF1R, or control shScramble. Scale bar, 200 μm. Right: the number of migrated cells was counted. Results are presented as mean ± SD from three independent experiments. *P < 0.05, **P < 0.01, ***P < 0.001. Two-tailed Student’s *t*-test

To explore the underlying mechanism of lnc-MLETA1-mediated cell motility, we performed RNA sequencing on lnc-MLETA1-knockdown and control CL1-5 cells. The heatmap showed the top 50 most increased and decreased genes among 778 differentially-expressed genes (fold change > 2). There are 315 upregulated genes and 463 downregulated genes in lnc-MLETA1-knockdown cells compared with control CL1-5 cells (Fig. 6A). Gene ontology (GO) analysis of the DEGs in lnc-MLETA1-knockdown cells showed 20-term enrichment, such as focal adhesion, integrin complex, and apical plasma membrane (Supplementary Fig. S6A). To further elucidate the effect of lnc-MLETA1 on global metastasis-associated gene changes, we performed gene set enrichment analysis (GSEA) on lnc-MLETA1-knockdown CL1-5 cells. GSEA indicated that three published metastasis-upregulated gene signatures were significantly enriched in downregulated genes (Fig. 6B). Conversely, another three published metastasis-downregulated gene signatures were significantly enriched in upregulated genes, strongly suggesting that lnc-MLETA1 is involved in the metastatic process and regulates the expression of metastasis-related genes. Furthermore, GSEA indicated that target gene signatures of miR-186-5p and miR-497-5p were significantly enriched in downregulated genes (Fig. 6C), suggesting that lnc-MLETA1 modulates the expression of the target genes of miR-186-5p and miR-497-5p that may support metastasis.

Recently, some studies have shown that lncRNAs share miRNAs with mRNAs and thus affect the expression of mRNAs by acting as competing endogenous RNAs [31]. Therefore, we performed a bioinformatic analysis starBase v3.0 to predict the target genes of miR-186-5p and miR-497-5p and to intersect the downregulated genes after silencing lnc-MLETA1 (Fig. 6D). Among these genes, epidermal growth factor receptor (EGFR) and insulin-like growth factor 1 receptor (IGF1R), which have been shown to be associated with lung cancer metastasis and progression [32–35], were selected for further studies. To verify that EGFR and IGF1R can be regulated by lnc-MLETA1, we performed qRT‒PCR analysis and western blot analysis to detect the expression of EGFR and IGF1R after silencing lnc-MLETA1. The results showed that knockdown of lnc-MLETA1 significantly decreased the mRNA and protein expression of EGFR and IGF1R compared with that in the control group (Fig. 6E-H). In addition, GSEA indicated that EGFR and IGF1R signaling-related gene signatures were significantly decreased after lnc-MLETA1 silencing (Supplementary Fig. S6B), suggesting that lnc-MLETA1 regulates the EGFR and IGF1R signaling pathways. To confirm that EGFR and IGF1R can be downregulated by miR-186-5p and miR-497-5p, respectively, we performed qRT‒PCR analysis and western blot analysis to detect the expression of EGFR and IGF1R after overexpression of miR-186-5p and miR-497-5p. The results showed that overexpression of miR-186-5p and miR-497-5p significantly decreased the mRNA and protein expression of EGFR and IGF1R compared with that of the control group, respectively (Fig. 6I-L). To further determine whether EGFR and IGF1R are the direct downstream targets of miR-186-5p and miR-497-5p, respectively, we predicted the binding sites of miR-186-5p on the EGFR 3'UTR and miR-497-5p on the IGF1R 3'UTR using starBase v3.0 and performed a luciferase reporter assay of CL1-0 cells transfected with miRNA mimics and pMIR-REPORT luciferase plasmids containing the 3'UTR of EGFR or IGF1R (Supplementary Fig. S6C and D). The results showed that overexpression of miR-186-5p and miR-497-5p significantly decreased the activity of reporter genes containing the 3'UTR of EGFR and IGF1R, respectively (Fig. 6M and N). These data suggested that EGFR and IGF1R are the direct downstream targets of miR-186-5p and miR-497-5p, respectively. To evaluate the contribution of lncRNA‒miRNA interactions to the expression of EGFR and IGF1R, we co-transfected CL1-5 cells with lnc-MLETA1 and miRNA mimics. Western blot analysis showed that overexpression of miR-186-5p and miR-497-5p significantly decreased the protein expression of EGFR and IGF1R compared with that of the control group, respectively, but it could be restored by overexpression of lnc-MLETA1 (Fig. 6O and P). To examine the effect of EGFR and IGF1R on lung cancer cell motility, we performed a wound-healing assay on CL1-5 cells infected with EGFR or IGF1R shRNAs. The results showed that knockdown of EGFR and IGF1R significantly decreased the number of migrated CL1-5 cells (Supplementary Fig. S6E-H). To further determine whether lnc-MLETA1 facilitates cell motility via EGFR and IGF1R, we performed a wound-healing assay on CL1-0 cells co-transfected with lnc-MLETA1 and shRNAs targeting EGFR or IGF1R. The results showed that overexpression of lnc-MLETA1 significantly increased the migration of CL1-0 cells, which was abolished by knockdown of EGFR and IGF1R (Fig. 6Q). In aggregate, these data suggested that lnc-MLETA1 promotes cell motility through the miR-186-5p/EGFR and miR-497-5p/IGF1R axes. In addition, to determine whether exosomes derived from CL1-5 cells promote cell migration through EGFR and IGF1R, we performed a wound-healing assay on CL1-0 cells incubated with exosomes derived from CL1-5 cells and transfected with shRNAs targeting EGFR or IGF1R. The results showed that exosomes derived from CL1-5 significantly increased the migration of CL1-0 cells, which was abolished by knockdown of EGFR and IGF1R (Supplementary Fig. S6I). These data suggested that the delivery of exosomes from high-metastatic cancer cells affects low-metastatic cancer migration by upregulating EGFR and IGF1R.

### Exosomal lnc-MLETA1 was correlated with tumor metastasis in lung cancer patients

**Fig. 7:**
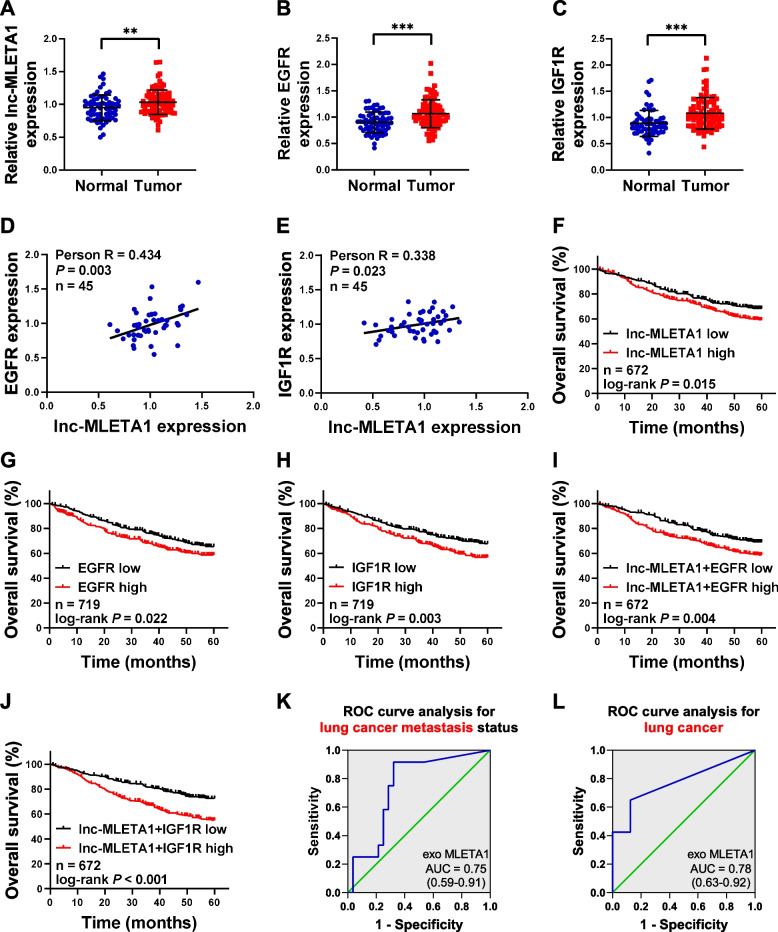
lnc-MLETA1 is upregulated in tumor tissues and predicts poor survival in lung cancer patients. **A-C** The expression of lnc-MLETA1 (**A**), EGFR (**B**) and IGF1R (**C**) between non-tumor and tumor tissue in the GSE19188 dataset. The data were analyzed with two-tailed Student’s *t*-test. **D** and **E** The Pearson correlation analysis of the expression of lnc-MLETA1 and EGFR (**D**) or IGF1R (**E**) in the GSE19188 dataset. **F–H** Kaplan–Meier analysis of overall survival in lung adenocarcinoma patients with high or low lnc-MLETA1 (**F**), EGFR (**G**) and IGF1R (**H**) expression. **I** and **J** Kaplan–Meier analysis of overall survival in lung adenocarcinoma patients with high- or low-scoring groups using the gene expression of lnc-MLETA1 plus EGFR (**I**) and lnc-MLETA1 plus IGF1R (**J**). The patients were divided into high and low groups based on the median expression value of the gene in the cohort and the data were analyzed with log-rank test. **K** and **L** ROC curve analysis of plasma exosomal lnc-MLETA1 for lung cancer metastasis (**K**) and lung cancer (**L**). Results are presented as mean ± SD. **P < 0.01, ***P < 0.001

We further examined the correlation of lnc-MLETA1 expression and the clinical outcome of lung cancer patients and found that the expression of lnc-MLETA1 was significantly higher in lung cancer tissues than in normal tissues in the Gene Expression Omnibus (GEO) dataset (Fig. 7A). The expression of EGFR and IGF1R was also significantly higher in lung cancer tissues than in normal tissues (Fig. 7B and C). Moreover, lnc-MLETA1 expression was positively correlated with EGFR and IGF1R expression in lung cancer tissues (Fig. 7D and E). Kaplan‒Meier analysis revealed that patients with high expression of lnc-MLETA1, EGFR and IGF1R had significantly poorer survival than patients with low expression (Fig. 7F-H). Furthermore, lung cancer patients with both elevated lnc-MLETA1 and EGFR or IGF1R displayed the worst survival, indicating the superior prognostic value of combining the two parameters compared with one gene alone (Fig. 7I and J). In addition, we examined the correlation between the expression of miR-186-5p and miR-497-5p and the clinical outcome of lung cancer patients. The results showed that the expression of miR-186-5p and miR-497-5p was significantly lower in lung cancer tissues than in normal tissues (Supplementary Fig. S7A and B). Moreover, miR-186-5p and miR-497-5p expression was negatively correlated with expression of the target genes EGFR and IGF1R in lung cancer tissues, respectively (Supplementary Fig. S7C and D). Kaplan‒Meier analysis indicated that patients with high expression of miR-186-5p and miR-497-5p had significantly better survival than patients with low expression (Supplementary Fig. S7E and F).

Notably, we detected exosomal lnc-MLETA1 levels isolated from plasma samples of lung cancer patients using qRT‒PCR analysis and found that elevated expression of exosomal lnc-MLETA1 was significantly associated with higher TNM stage and metastasis in lung cancer patients (Table 1). In addition, the levels of exosomal lnc-MLETA1 in the plasma of lung cancer patients were significantly higher than those in the plasma of healthy subjects (Supplementary Table S1). Receiver operating characteristic (ROC) analysis showed that plasma exosomal lnc-MLETA1 could be a diagnostic biomarker for lung cancer metastasis and discriminated lung cancer patients from healthy subjects (Fig. 7K and L). Intriguingly, we detected the expression of lnc-MLETA1 in the primary and metastatic tissues of lung cancer patients using in situ hybridization staining and found that the levels of lnc-MLETA1 in metastatic tissues were obviously higher than those in primary tissues (Supplementary Fig. S7G).

**Table 1:** Correlations between exosomal lnc-MLETA1 expression and clinicopathological features

Variables	Low exo MLETA1 (*n* = 20)	High exo MLETA1 (*n* = 20)	*P* value
**Gender**			0.749
Male	9	8	
Female	11	12	
**Age**			0.705
< 60	5	4	
≥ 60	15	16	
**Tumor size (cm)**			0.204
< 3	11	7	
≥ 3	9	13	
**Recurrence**			0.519
No	7	9	
Yes	13	11	
**TNM stage**			0.011*
I/II	13	5	
III/IV	7	15	
**Metastasis**			< 0.001*
No	19	9	
Yes	1	11	

The expression level of exosomal lnc-MLETA1 was examined by RT-qPCR. The patients were split by the median concentration of lnc-MLETA1 and the data were analyzed with Chi-squared test. *P* values < 0.05 were considered statistically significant

**Fig. 8:**
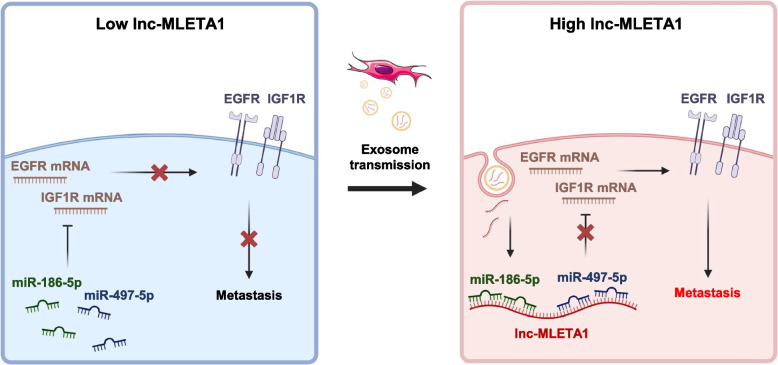
Schematic diagram of lnc-MLETA1-based regulatory mechanism in lung cancer metastasis. Exosomal lnc-MLETA1 can be delivered from high metastatic lung cancer cells to low metastatic lung cancer cells. Moreover, lnc-MLETA1 regulates the expression of EGFR and IGF1R by sponging miR-186-5p and miR-497-5p, leading to lung cancer cell motility and metastasis

In conclusion, lnc-MLETA1 is upregulated in highly metastatic cancer cells and their secreted exosomes and predicts the survival of lung cancer patients. Mechanistically, lnc-MLETA1 promotes cell motility and regulates the expression of EGFR and IGF1R by sponging miR-186-5p and miR-497-5p. Our findings suggest that lnc-MLETA1 plays a critical role in lung cancer metastasis and may serve as a biomarker for lung cancer diagnosis and prognosis and a potential target for lung cancer treatment (Fig. 8).

## Discussion

In recent years, growing evidence has shown that tumor-derived exosomes promote lung cancer metastasis [6, 36]. Although researchers have revealed that exosomal miRNAs or proteins can facilitate lung cancer cell motility and tumor metastasis, the effect of exosomal lncRNAs on lung cancer metastasis remains poorly understood. In the present study, we first identified a novel lncRNA, MLETA1, that was elevated in highly metastatic lung cancer cells and their secreted exosomes. Moreover, lnc-MLETA1 was required for lung cancer cell motility and metastasis. Importantly, lnc-MLETA1 could be packaged into exosomes and disseminated to poorly metastatic cells, leading to lung cancer metastasis. Furthermore, lnc-MLETA1 modulated the expression of EGFR and IGF1R by competitively binding miR-186-5p and miR-497-5p. In addition, we verified that lnc-MLETA1 was upregulated in tumor tissues and correlated with poor survival in lung cancer patients. Interestingly, exosomal lnc-MLETA1 detected in plasma from lung cancer patients was associated with lung cancer metastasis. Our findings indicate that exosomal lnc-MLETA1 may be a prognostic indicator for metastatic lung cancer and may serve as a therapeutic target for lung cancer treatment.

Extensive evidence indicates that the nuclear or cytoplasmic localization of lncRNAs is associated with the cellular functions of lncRNAs [17]. lncRNAs can interact with miRNAs, mRNAs, and proteins to regulate post-transcriptional modification in the cytoplasm [37, 38]. However, lncRNAs recruit transcription factors and bind promoter regions to modulate gene transcription in the nucleus [39]. Hence, we performed subcellular fractionation to demonstrate that lnc-MLETA1 in lung cancer cells is mainly located in the cytoplasm. lnc-MLETA1 acts as a competitive endogenous RNA to sponge miRNAs and influences the expression of downstream targets of miRNAs. Precursor miRNAs are processed to mature miRNA by Drosha and Dicer and retained in the RNA-induced silencing complex (RISC), which contains the key protein Argonaute 2 (Ago2) [40]. Therefore, an MS2-tagged RNA affinity purification (MS2-TRAP) assay was used to confirm the presence of Ago2 in the MS2-lnc-MLETA1 pulldown samples and demonstrate that lnc-MLETA1 might interact with miRNAs. In parallel, we further verified that lnc-MLETA1 interacts with miR-186-5p and miR-497-5p. Apart from miRNAs, whether lnc-MLETA1 also binds to mRNAs or proteins to promote lung cancer metastasis and progression requires further study. In the future, we will explore the other regulatory mechanisms of lnc-MLETA1 in lung cancer.

Previous studies indicated that EGFR and IGF1R are essential in lung cancer progression [34, 41–43]. Overexpression of EGFR and IGF1R induces lung cancer cell proliferation, migration, invasion, drug resistance, stemness and tumor metastasis. The clinical data revealed that the expression of EGFR and IGF1R is associated with poor outcomes in lung cancer patients. Interestingly, our findings showed that miR-186-5p and miR-497-5p modulate the expression of EGFR and IGF1R by directly binding their 3'UTRs, respectively. Recently, some studies have shown that miR-186-5p and miR-497-5p inhibit lung cancer cell growth, migration, and invasion [44, 45]. Moreover, the expression of miR-186-5p and miR-497-5p was negatively correlated with TNM stage, lymph node metastasis and survival in lung cancer patients, suggesting that miR-186-5p and miR-497-5p are tumor suppressor miRNAs in lung cancer. Importantly, we found that lnc-MLETA1 regulated the expression of EGFR and IGF1R by sponging miR-186-5p and miR-497-5p to facilitate cell motility. Thus, these data indicated that lnc-MLETA1 plays a crucial role in lung cancer progression and metastasis.

Antisense oligonucleotide (ASO)-based therapeutics are a promising novel strategy against various diseases, including hypercholesterolemia, Huntington's disease, Alzheimer's disease, Parkinson's disease, Duchenne muscular dystrophy, and cancer [46–48]. ASO drugs are single-stranded chains of synthetic oligonucleotides that specifically bind a target RNA and block its function through Watson–Crick base pairing [49]. As of 2022, the U.S. Food and Drug Administration (FDA) already approved 10 ASO drugs [50]. Although these 10 ASO drugs are not for cancer, other anticancer ASO candidates have entered clinical development [51]. Moreover, researchers are still investigating the underlying mechanisms of lncRNA-mediated tumor progression and attempting to design specific locked nucleic acid antisense oligonucleotides (LNA ASOs) to target lncRNAs. For example, Qu et al. found that targeting lncARSR with LNA ASO-based treatment overcame drug resistance in advanced renal cell carcinoma [31]. Tan et al. found that targeting EGFR-AS1 with LNA induced tumor regression in squamous cell carcinoma [52]. In our study, we designed an LNA against lnc-MLETA1 and found that specific targeting of lnc-MLETA1 suppressed highly metastatic lung cancer cell-derived exosome-mediated cell migration and invasion. In the future, we will further examine the effect of LNA or other strategies targeting lnc-MLETA1 on lung cancer metastasis in vivo, hoping that lncRNA-targeted therapy could be applied as a novel therapeutic strategy for lung cancer treatment.

## Conclusions

This study identifies lnc-MLETA1 as a critical exosomal lncRNA that mediates crosstalk in lung cancer cells to promote cancer metastasis and may serve as a prognostic biomarker and potential therapeutic target for lung cancer diagnosis and treatment.

### Supplementary Information

**Additional file 1: Supplementary Figure 1.** Characterization of lnc-MLETA1. **Supplementary Figure 2.** Knockdown of lnc-MLETA1 suppresses cell growth and anchorage-independent growth ability of lung cancer cell. **Supplementary Figure 3.** Exosome-transmitted lnc-MLETA1 is uptake by CL1-0 cells. **Supplementary Figure 4.** Exosomal lnc-MLETA1 augments tumor growth in vivo. **Supplementary Figure 5.** Regulatory relationships between lnc-MLETA1 and miR-186-5p or miR-497-5p. **Supplementary Figure 6.** Knockdown of EGFR and IGF1R attenuates lung cancer cell migration. **Supplementary Figure 7.** miR-186-5p and miR-497-5p are downregulated in tumor tissues and predicts good survival in lung cancer patients. **Supplementary Table S1.** Correlations between exosomal lnc-MLETA1 expression and lung cancer diagnosis. **Supplementary Table S2.** Sequences of primers/shRNA/LNA used in this study.

## Data Availability

All data generated or analyzed during this study are included in this published article and its supplementary information files.

## Abbreviations

lncRNA: Long noncoding RNA
NSCLC: Non-small cell lung cancer
RNA-seq: RNA sequencing
qRT-PCR: Quantitative real-time polymerase chain reaction
MS2-TRAP: MS2-tagged RNA affinity purification assay
ceRNA: Competing endogenous RNA
EGFR: Epidermal growth factor receptor
IGF1R: Insulin like growth factor 1 receptor
