# Supplementary material for: Exosomal long noncoding RNA MLETA1 promotes tumor progression and metastasis by regulating the miR-186-5p/EGFR and miR-497-5p/IGF1R axes in non-small cell lung cancer
Source: J Exp Clin Cancer Res. 2023 Oct 26;42:283. doi: 10.1186/s13046-023-02859-y (PMC10601119; doi:10.1186/s13046-023-02859-y)
Supplement: Supplementary file 1 — Additional file 1: Supplementary Figure 1. Characterization of lnc-MLETA1. Supplementary Figure 2. Knockdown of lnc-MLETA1 suppresses cell growth and anchorage-independent growth ability of lung cancer cell. Supplementary Figure 3. Exosome-transmitted lnc-MLETA1 is uptake by CL1-0 cells. Supplementary Figure 4. Exosomal lnc-MLETA1 augments tumor growth in vivo. Supplementary Figure 5. Regulatory relationships between lnc-MLETA1 and miR-186-5p or miR-497-5p. Supplementary Figure 6. Knockdown of EGFR and IGF1R attenuates lung cancer cell migration. Supplementary Figure 7. miR-186-5p and miR-497-5p are downregulated in tumor tissues and predicts good survival in lung cancer patients. Supplementary Table S1. Correlations between exosomal lnc-MLETA1 expression and lung cancer diagnosis. Supplementary Table S2. Sequences of primers/shRNA/LNA used in this study. [file 13046_2023_2859_MOESM1_ESM.pdf]

Figure S1

A

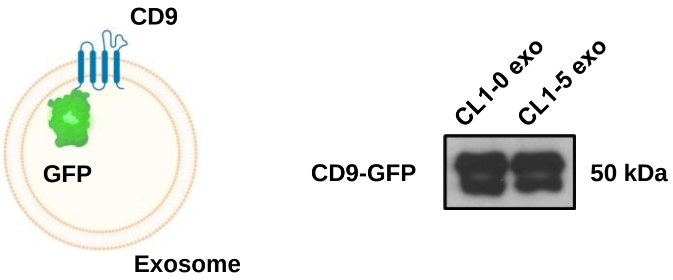

C

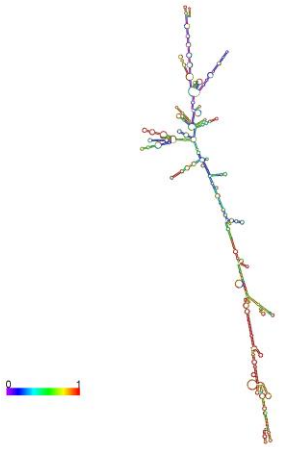

B

CPAT
Coding Potential Assessment Tool

Calculator User Guide Feedback Source Code

Result for species name : hg19 with job ID :1691114637							
Data ID	Sequence Name	RNA Size	ORF Size	Ficket Score	Hexamer Score	Coding Probability	Coding Label
0	NR_040000.1	1821	294	0.7384	-0.230624462663	0.014322511671243	no

D

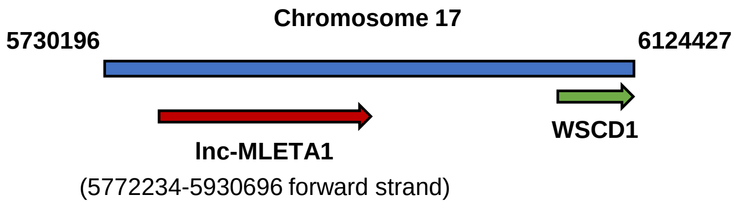

E

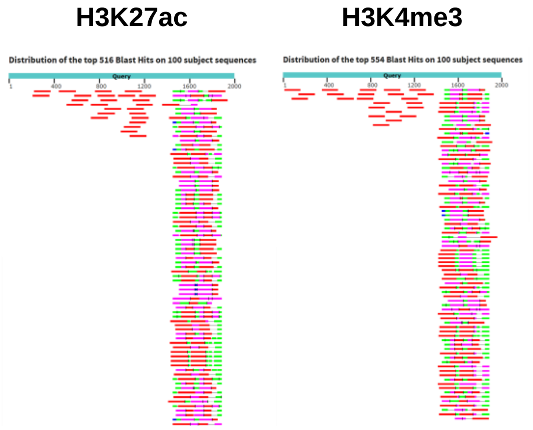

F

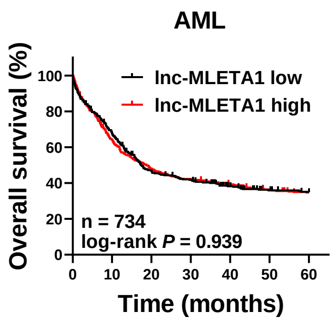

G

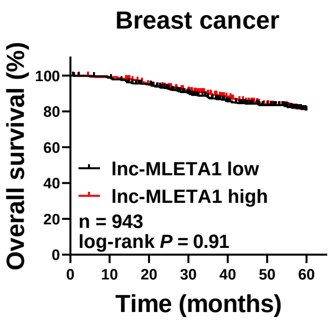

H

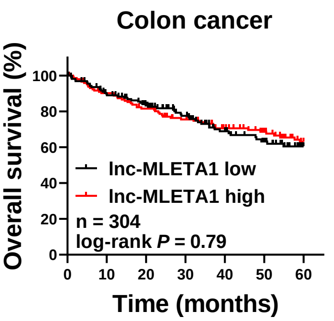

I

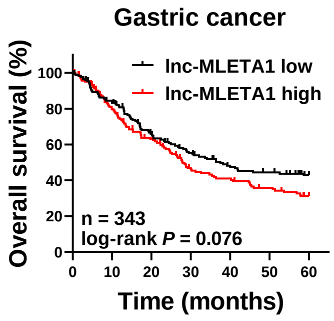

J

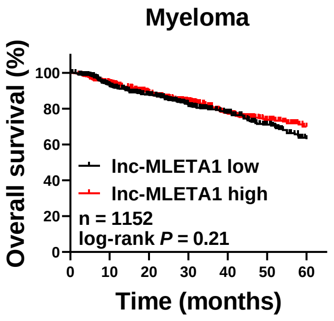

K

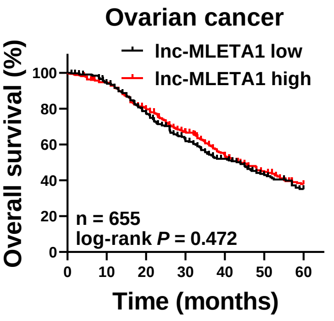

Supplementary Figure 1. Characterization of lnc-MLETA1. A Western blot analysis of GFP expression in exosomes derived from CL1-0 and CL1-5 cells transiently transfected with pEGFP-N-CD9 plasmids. **B** The sequence of lnc-MLETA1 was identified without protein-coding potential by Coding Potential Assessment Tool (CPAT). **C** The secondary structure of lnc-MLETA1 was predicted by RNAfold web server. **D** Schematic annotation of the lnc-MLETA1 genomic locus on chromosome 17: 5772234-5930696 forward strand. **E** The enrichment of active histone markers H3K27ac and H3K4me3, within the genomic regions encompassing lnc-MLETA1 in the GSE225332 dataset. **F-K** Kaplan–Meier analysis of overall survival in cancer patients, including AML (**F**), breast cancer (**G**), colon cancer (**H**), gastric cancer (I), myeloma (**J**), and ovarian cancer (**K**) with high or low lnc-MLETA1 expression. The patients were divided into high and low groups based on the median expression value of the gene in the cohort and the data were analyzed with log-rank test.

Figure S2

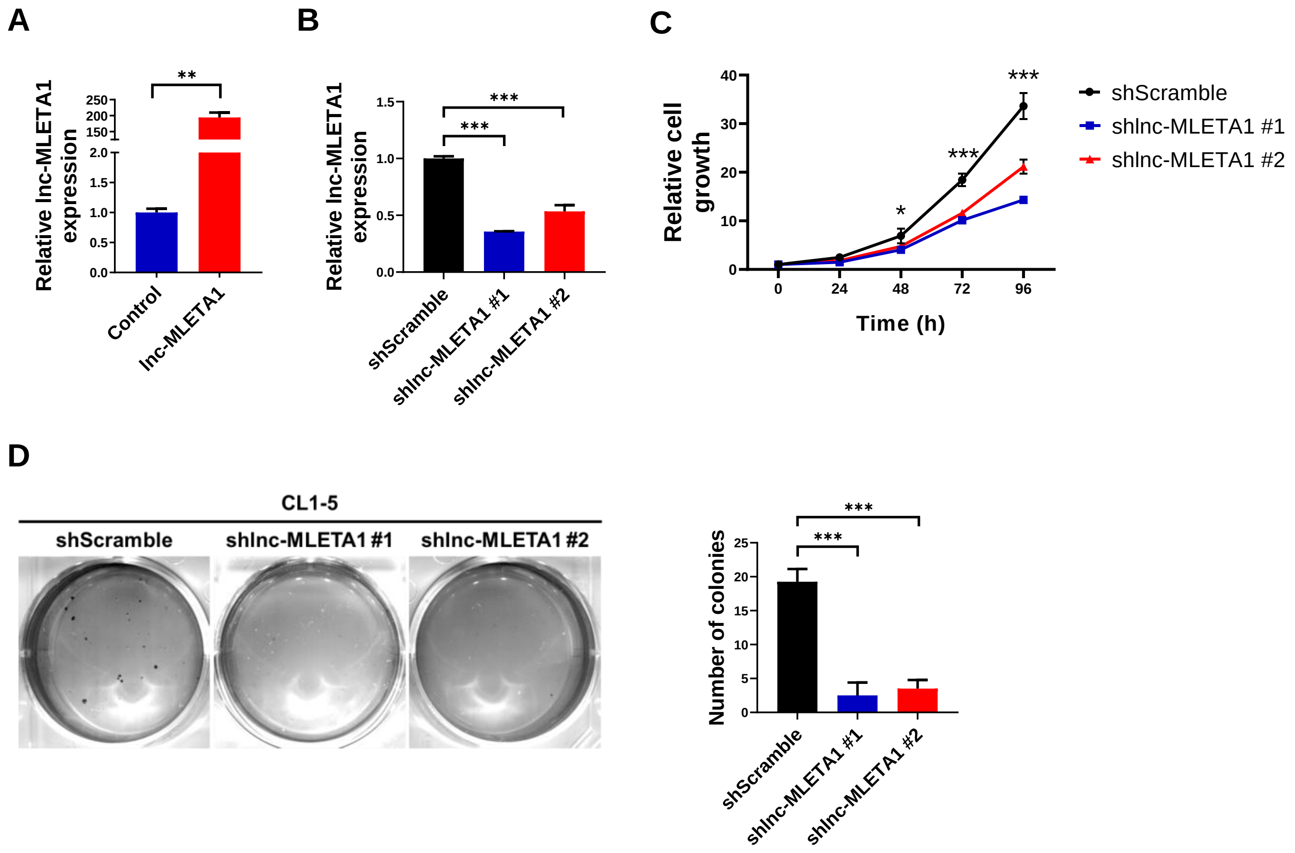

Supplementary Figure 2. Knockdown of lnc-MLETA1 suppresses cell growth and anchorage-independent growth ability of lung cancer cell. A qRT-PCR analysis of lnc-MLETA1 expression in CL1-0 cells transfected with lnc-MLETA1 plasmids or control plasmids. **B** qRT-PCR analysis of lnc-MLETA1 expression in CL1-5 cells infected with shlnc-MLETA1 virus or control virus. **C** Relative cell growth of lnc-MLETA1-knockdown and control CL1-5 cells was analyzed with a WST-1 assay at indicated times. **D** Soft agar colony formation assay of lnc-MLETA1-knockdown and control CL1-5 cells for 2 weeks. Left: representative images of colonies. Right: the number of colonies was calculated. Results are presented as mean ± SD from three independent experiments. $^{*}P < 0.05$, $^{**}P < 0.01$, $^{***}P < 0.001$. Two-tailed Student's *t*-test.

Figure S3

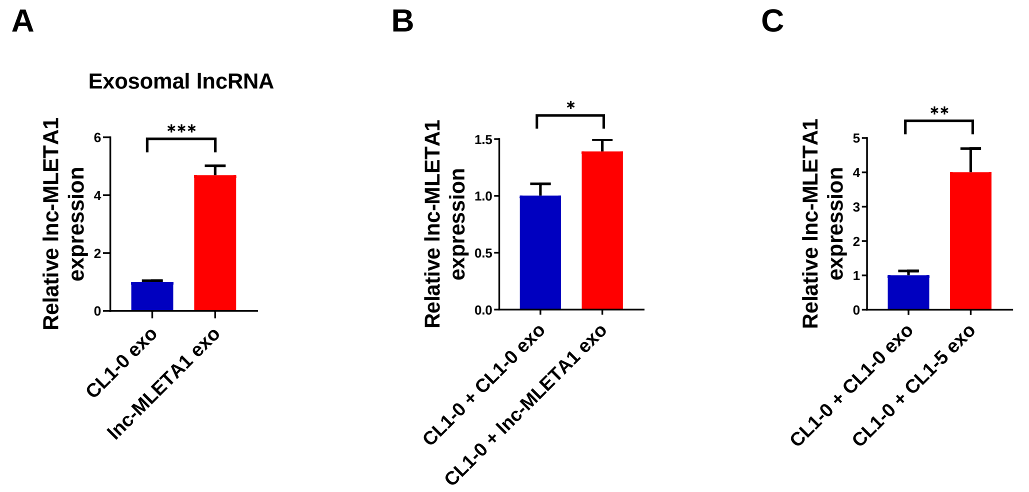

Supplementary Figure 3. Exosome-transmitted lnc-MLETA1 is uptake by CL1-0 cells. A qRT-PCR analysis of lnc-MLETA1 levels in exosomes derived from lnc-MLETA1-overexpressing and control CL1-0 cells. **B** qRT-PCR analysis of lnc-MLETA1 levels in CL1-0 cells pre-incubated with exosomes derived from lnc-MLETA1-overexpressing or control CL1-0 cells for 48 h. **C** qRT-PCR analysis of lnc-MLETA1 levels in CL1-0 cells pre-incubated with exosomes derived from CL1-0 or CL1-5 cells for 48 h. Results are presented as mean ± SD from three independent experiments. *P < 0.05, **P < 0.01, ***P < 0.001. Two-tailed Student's t-test.

Figure S4

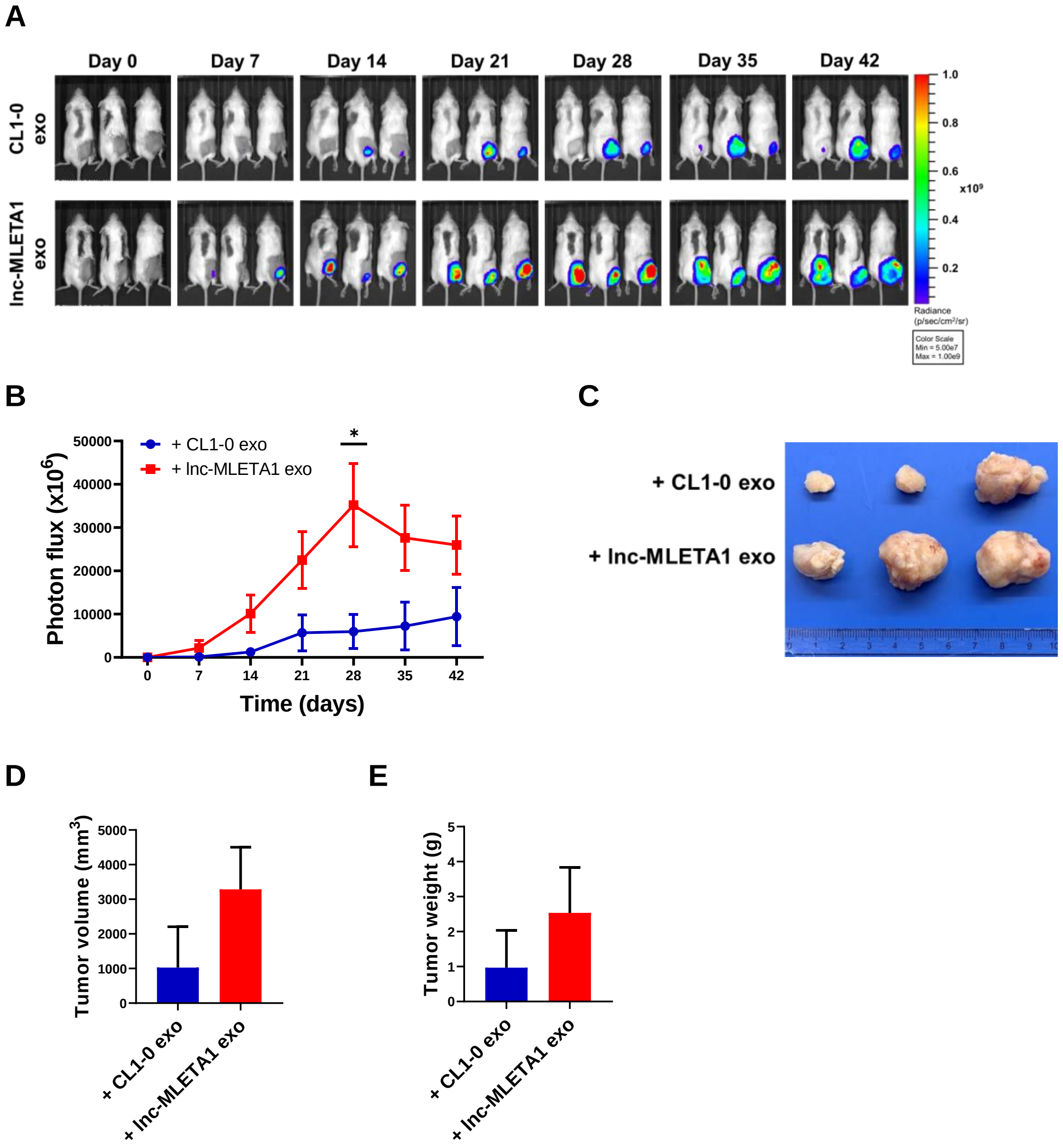

Supplementary Figure 4. Exosomal lnc-MLETA1 augments tumor growth *in vivo*. A-E NOD-SCID mice were subcutaneously xenografted with CL1-0 cells and injected intratumorally with exosomes derived from lnc-MLETA1-overexpressing or control CL1-0 cells twice a week. Representative bioluminescent images (**A**), quantification of bioluminescent imaging signal intensities (**B**), representative images of subcutaneous xenografts (**C**), tumor volumes (**D**), and tumor weight (**E**) are shown. Results are presented as mean ± SD from three independent experiments. *$P < 0.05$. Two-tailed Student's *t*-test.

Figure S5

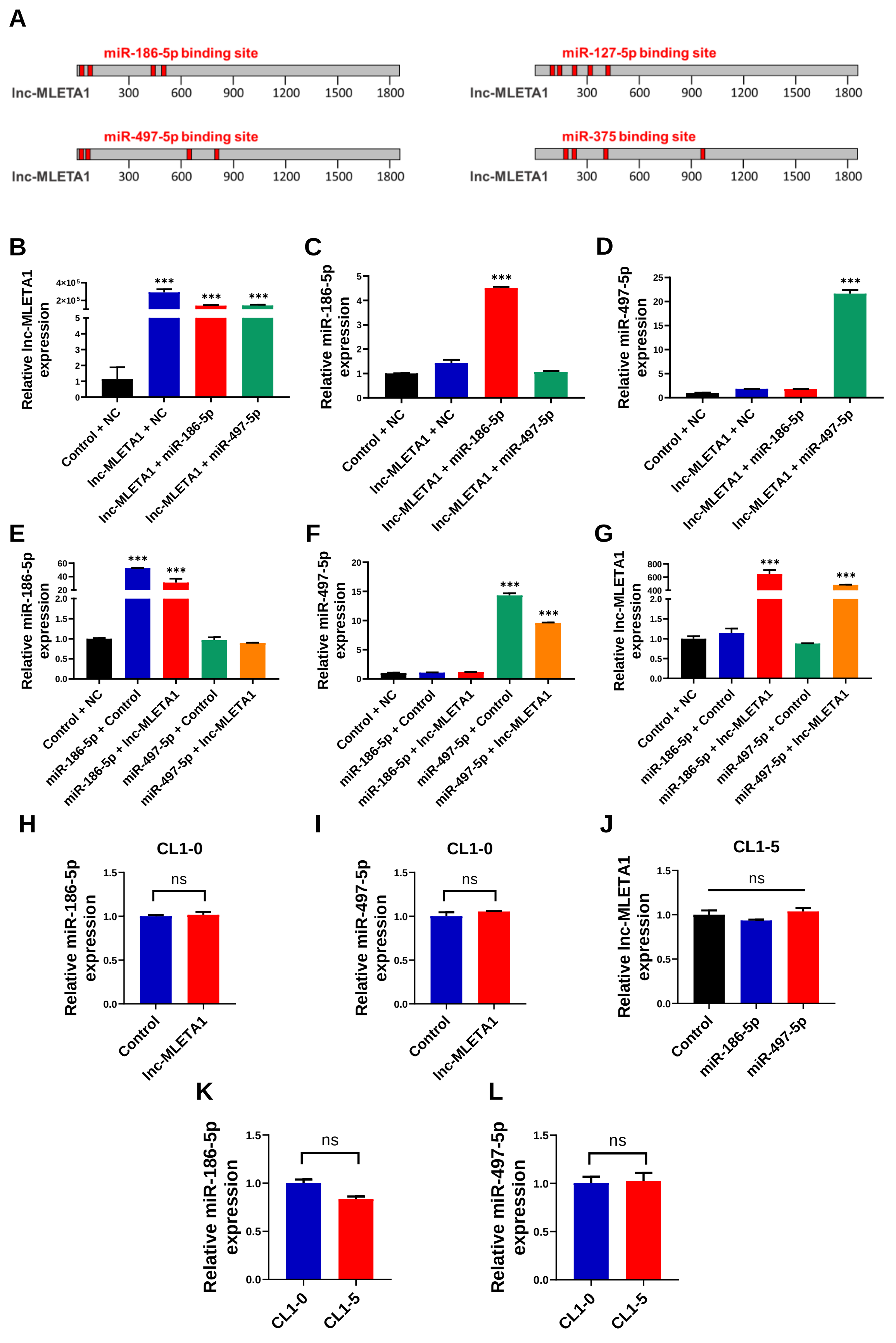

A
miR-186-5p binding site
miR-127-5p binding site
miR-497-5p binding site
miR-375 binding site
lnc-MLETA1
300
600
900
1200
1500
1800
B
C
D
E
F
G
Relative lnc-MLETA1 expression
Relative miR-186-5p expression
Relative miR-497-5p expression
Control + NC
lnc-MLETA1 + NC
lnc-MLETA1 + miR-186-5p
lnc-MLETA1 + miR-497-5p
miR-186-5p + Control
miR-186-5p + lnc-MLETA1
miR-497-5p + Control
miR-497-5p + lnc-MLETA1
***
H
I
J
K
L
CL1-0
CL1-5
ns
Control
lnc-MLETA1
miR-186-5p
miR-497-5p

Supplementary Figure 5. Regulatory relationships between lnc-MLETA1 and miR-186-5p or miR-497-5p. A Schematic of predicted binding sites of miR-186-5p and miR-497-5p on lnc-MLETA1. **B-D** qRT-PCR analysis of lnc-MLETA1 (**B**), miR-186-5p (**C**), and miR-497-5p (**D**) expression in CL1-0 cells co-transfected with lnc-MLETA1 plasmids or control plasmids and with miRNA mimics or negative control. **E-G** qRT-PCR analysis of miR-186-5p (**E**), miR-497-5p (**F**), and lnc- MLETA1 (**G**) expression in CL1-5 cells co-transfected with lnc-MLETA1 plasmids or control plasmids and with miRNA mimics or negative control. **H** and **I** qRT-PCR analysis of miR-186-5p (**H**) and miR-497-5p (**I**) expression in CL1-0 cells transfected with lnc-MLETA1 plasmids or control plasmids. **J** qRT-PCR analysis of lnc-MLETA1 expression in CL1-5 cells transfected with miR-186-5p, miR-497-5p or negative control. **K** and **L** qRT-PCR analysis of miR-186-5p (**K**) and miR-497-5p (**L**) expression in CL1-0 and CL1-5 cells. Results are presented as mean ± SD from three independent experiments. ***$P < 0.001$. Two-tailed Student's t-test.

Figure S6

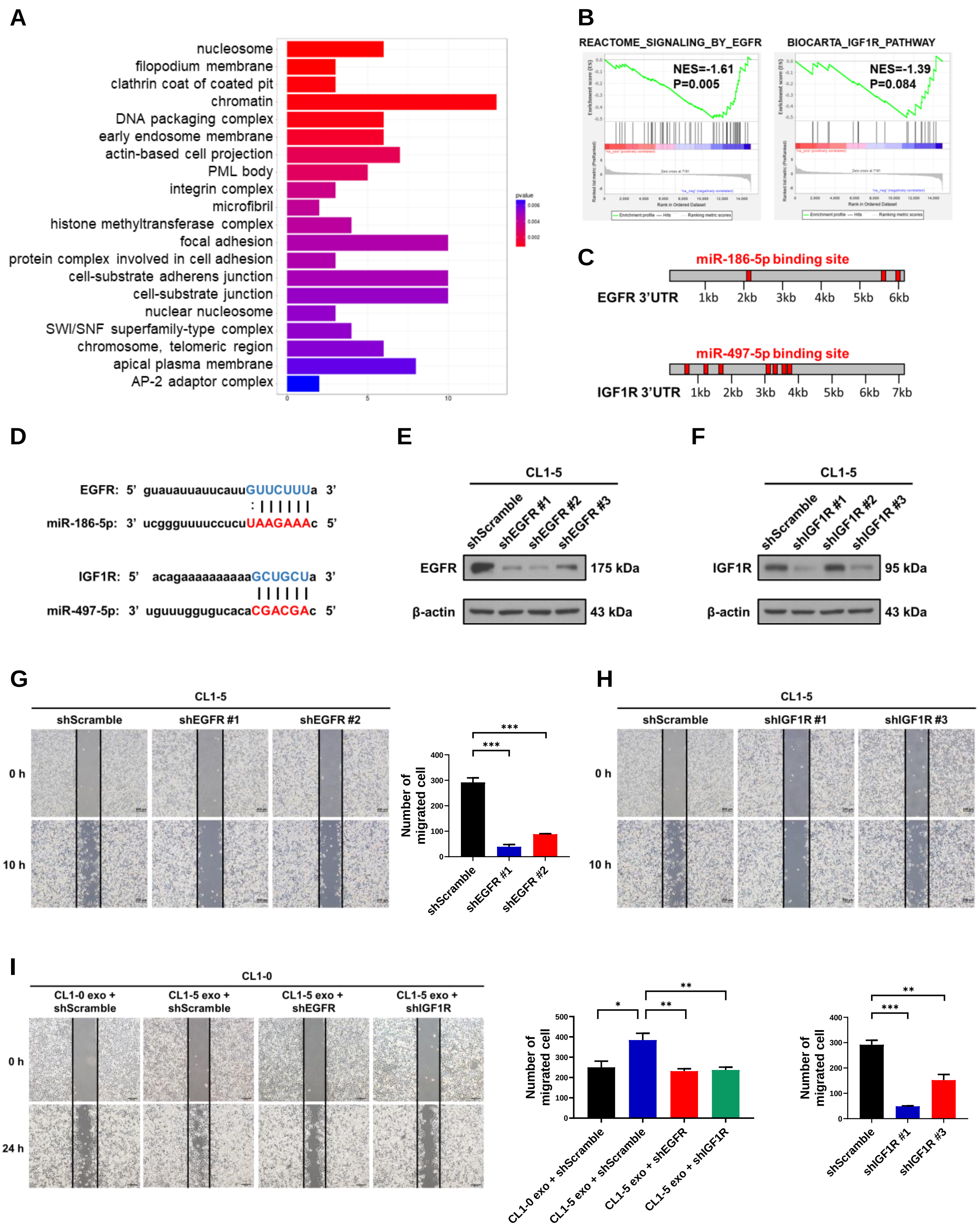

A
nucleosome
filopodium membrane
clathrin coat of coated pit
chromatin
DNA packaging complex
early endosome membrane
actin-based cell projection
PML body
integrin complex
microfibril
histone methyltransferase complex
focal adhesion
protein complex involved in cell adhesion
cell-substrate adherens junction
cell-substrate junction
nuclear nucleosome
SWI/SNF superfamily-type complex
chromosome, telomeric region
apical plasma membrane
AP-2 adaptor complex
pvalue
B
REACTOME_SIGNALING_BY_EGFR
NES=-1.61
P=0.005
BIOCARTA_IGF1R_PATHWAY
NES=-1.39
P=0.084
C
miR-186-5p binding site
EGFR 3'UTR 1kb 2kb 3kb 4kb 5kb 6kb
miR-497-5p binding site
IGF1R 3'UTR 1kb 2kb 3kb 4kb 5kb 6kb 7kb
D
EGFR: 5' guauauuauucauuGUUCUUUa 3'
miR-186-5p: 3' ucggguuuuccucuUAAGAAAc 5'
IGF1R: 5' acagaaaaaaaaaaGCUGCUa 3'
miR-497-5p: 3' uguuuggugucacaCGACGAc 5'
E
CL1-5
shScramble shEGFR #1 shEGFR #2 shEGFR #3
EGFR 175 kDa
β-actin 43 kDa
F
CL1-5
shScramble shIGF1R #1 shIGF1R #2 shIGF1R #3
IGF1R 95 kDa
β-actin 43 kDa
G
CL1-5
shScramble shEGFR #1 shEGFR #2
0 h
10 h
Number of migrated cell
***
***
shScramble shEGFR #1 shEGFR #2
H
CL1-5
shScramble shIGF1R #1 shIGF1R #3
0 h
10 h
I
CL1-0
CL1-0 exo + shScramble
CL1-5 exo + shScramble
CL1-5 exo + shEGFR
CL1-5 exo + shIGF1R
0 h
24 h
Number of migrated cell
*
**
**
CL1-0 exo + shScramble
CL1-5 exo + shScramble
CL1-5 exo + shEGFR
CL1-5 exo + shIGF1R
Number of migrated cell
***
**
shScramble shIGF1R #1 shIGF1R #3

Supplementary Figure 6. Knockdown of EGFR and IGF1R attenuates lung cancer cell migration. A Gene ontology (GO) analysis of the DEGs in lnc-MLETA1-knockdown cells versus control cells. **B** Gene Set Enrichment Analysis (GSEA) of published EGFR and IGF1R signaling pathway signatures in lnc-MLETA1-knockdown cells versus control cells. **C and D** Schematic of predicted binding sites of miR-186-5p on EGFR 3'UTR and miR-497-5p on IGF1R 3'UTR. **E** Western blot analysis of EGFR expression of EGFR-knockdown and control CL1-5 cells. **F** Western blot analysis of IGF1R expression of IGF1R-knockdown and control CL1-5 cells. **G** Left: representative images of wound-healing assay of EGFR-knockdown and control CL1-5 cells for 10 h. Scale bar, 200μm. Right: the number of migrated cells was calculated. **H** Upper: representative images of wound-healing assay of IGF1R-knockdown and control CL1-5 cells for 10 h. Scale bar, 200μm. Lower: the number of migrated cells was calculated. **I** Left: representative images of wound-healing assay of CL1-0 cells pre-incubated with CL1-0 or CL1-5 exosomes and transfected with shEGFR, shIGF1R, or control shScramble for 48 h. Scale bar, 200μm. Right: the number of migrated cells was counted. Results are presented as mean ± SD from three independent experiments. $*P < 0.05$, $**P < 0.01$, $***P < 0.001$. Two-tailed Student's *t*-test.

Figure S7

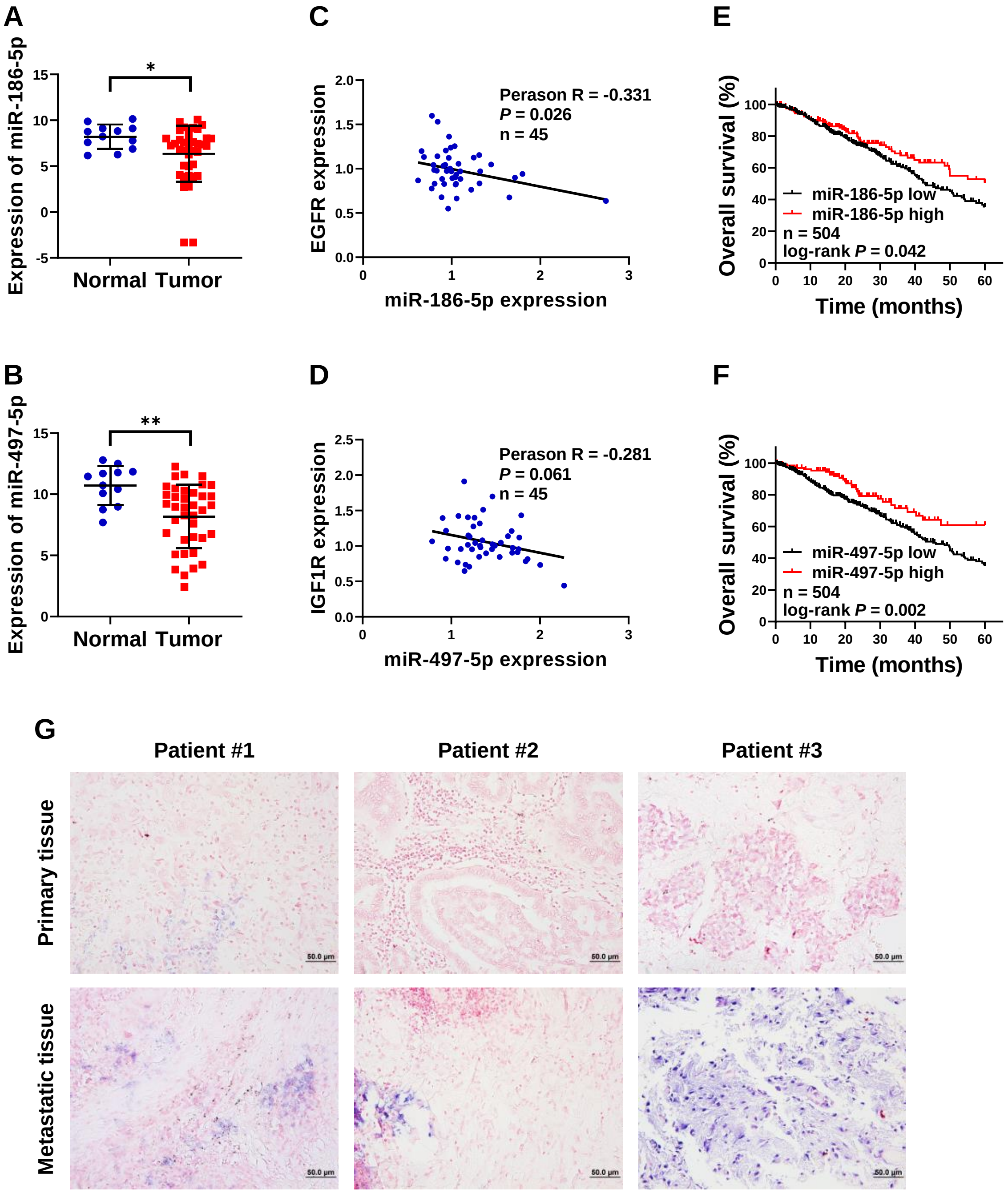

Supplementary Figure 7. miR-186-5p and miR-497-5p are downregulated in tumor tissues and predicts good survival in lung cancer patients. A and B The expression of miR-186-5p (**A**) and miR-497-5p (**B**) between non-tumor and tumor tissue in the GSE169587 dataset. The data were analyzed with two-tailed Student's *t*-test. **C** The Pearson correlation analysis of the expression of miR-186-5p and EGFR in the GSE19188 dataset. **D** The Pearson correlation analysis of the expression of miR-497-5p and IGF1R in the GSE19188 dataset. **E and F** Kaplan–Meier analysis of overall survival in lung adenocarcinoma patients with high or low miR-186-5p (**E**) and miR-497-5p (**F**) expression. The patients were split by the auto-selected best cutoff and the data were analyzed with log-rank test. **G** Representative microscopic images of *in situ* hybridization (ISH) staining in the primary and metastatic tissue. Scale bar, 50 μm. Results are presented as mean ± SD. *$P < 0.05$, **$P < 0.01$.

Table S1

Supplementary Table S1 Correlations between exosomal lnc-MLETA1 expression and lung cancer diagnosis.

Variables	Low exo MLETA1 (*n* = 24)	High exo MLETA1 (*n* = 24)	*P* value
**Diagnosis**			0.02*
Normal	7	1	
Tumor	17	23	

The expression level of exosomal lnc-MLETA1 was examined by RT-qPCR. The subjects were split by the median concentration of lnc-MLETA1 and the data were analyzed with Chi-squared test. *P* values < 0.05 were considered statistically significant.

Table S2

Supplementary Table S2 Sequences of primers/shRNA/LNA used in this study.

**qRT-PCR**	lnc-MLETA1	Forward	CTAGGGCTCTCCTGGCTGTA
		Reverse	TGCAACTTGAGGCAACAACG
	lnc-OR8D4-4	Forward	TGGAGTCTTGGCAGCTGATG
		Reverse	GGAGGGCTGAAGAACGACAA
	lnc-DLK1-35	Forward	GGGCATTAAGCCCTGACCTT
		Reverse	CCTTGGGGAGGGAAACACTC
	LINC00665	Forward	GTGTGAGTCCTCAGTCTTGGG
		Reverse	CCGGTGGACGGATGAGAAAC
	LINC00189	Forward	GGCCTTGGAGAGAAAACTTGC
		Reverse	CAGGCCAAAAGCTGGCAAAG
	IER3-AS1	Forward	CCCTTCTTGAGCCGGGAATC
		Reverse	TGAAGTCGCCTTTAGGGTGG
	lnc-RSF1-1	Forward	CACCGGCTGAGTGGATTCAA
		Reverse	AGGAGTGGAGATGACAGGCT
	lnc-TNS4-4	Forward	GCTCCGGTCTCGAATTTTGG
		Reverse	TGGTAAGGTACCCCTGGCA
	lnc-SPIN1-1:4	Forward	GAAGAGTCATGGCCTTGGT
		Reverse	CAGGAGCAGTGGTTCAGTATG
	lnc-SPIN1-1:2	Forward	ATTCATCCAGCAACCCTCTT
		Reverse	GGATGTGTCTGTGAAGGTGTTT
	lnc-ZNF37A-19	Forward	GGATTGTAATGGAAAGATATCAAATG
		Reverse	CCAGTCCATTCCAATTGATTC
	lnc-ZNF611-1	Forward	GAACAACAGGATGAGTGGTTTG
		Reverse	GGCTACTTGAACTGTGCATGTC
	lnc-CMTM3-1	Forward	CCTAGACAACCTGGTAACTACCAAC
		Reverse	CAAGGAGACCGGTTGGATTT
	lnc-ZNF66-9	Forward	TCAGGAAGCACGTTTCAGG
		Reverse	TTGTCCTCAGCAGCAGCTT
	EGFR	Forward	TGAGCTCTCTGAGTGCAACC
		Reverse	GTGGGGTCTGAGCTGTATCG
	IGF1R	Forward	TGTCCAGGCCAAAACAGGAT
		Reverse	CATTCCCCAGCCTGCTGTTA
	GAPDH	Forward	TGAAGGTCGGAGTCAACGGATT
		Reverse	CCTGGAAGATGGTGATGGGATT
	18S rRNA	Forward	GTAACCCGTTGAACCCCATT
		Reverse	CCATCCAATCGGTAGTAGCG
**shRNA**	shScramble		CCTAAGGTTAAGTCGCCCTCG
	shlnc-MLETA1 #1		GCACTCTCTCTCTCTGAATCC
	shlnc-MLETA1 #2		GCTGGAGGCTGGAGCTCTAAG
	shEGFR #1		GAGAATGTGGAATACCTAAGG
	shEGFR #2		GCCACAAAGCAGTGAATTTAT
	shEGFR #3		CCTCCAGAGGATGTTCAATAA
	shIGF1R #1		CATGTACTGCATCCCTTGTGA
	shIGF1R #2		GAGACAGAGTACCCTTTCTTT
	shIGF1R #3		CTTCGAGATGACCAATCTCAA
**LNA**	lnc-MLETA1 LNA		GAGGTAGGAGGCGTGG
